# Ras protein activator-like 3 is a key Ras GTPase-activating protein that protects the survival and anti-tumor activity of CD8^+^ T cells

**DOI:** 10.3389/fimmu.2026.1893111

**Published:** 2026-09-16

**Authors:** Zhenzi Peng, Xinyi Zhou, Alato Okuno, Derick Okwan-Duodu, Suguru Saito

**Affiliations:** 1 Department of Cell Biology and Genetics, School of Basic Medical Sciences, Hengyang Medical School, University of South China, Hengyang, Hunan, China; 2 Department of Health and Nutrition, Faculty of Human Design, Shibata Gakuen University, Hirosaki, Aomori, Japan; 3 Department of Pathology, School of Medicine, Stanford University, Stanford, CA, United States; 4 Department of Biomedical Sciences, Cedars-Sinai Medical Center, Los Angeles, CA, United States; 5 Department of Pathology and Laboratory Medicine, Cedars-Sinai Medical Center, Los Angeles, CA, United States

**Keywords:** anti-tumor immunity, CD8^+^ T cells, melanoma, RASAL3, RasGAP

## Abstract

Ras signaling plays an essential role in immune cell function; however, its activity must be precisely controlled to prevent excessive activation and associated cellular dysfunction. In this study, we identify the leukocyte-specific Ras GTPase-activating protein (RasGAP) RASAL3 as an important regulator of CD8^+^ T cell homeostasis and anti-tumor immunity. Following T cell receptor (TCR) stimulation, RASAL3-deficient CD8^+^ T cells display sustained Ras activation, resulting in increased activation-induced cell death (AICD). The surviving RASAL3-deficient CD8^+^ T cells exhibit impaired effector functions, including reduced cytokine production and decreased tumor-killing activity. Using an *in vivo* B16-F10 melanoma model, we found that CD8^+^ T cell-specific RASAL3-deficient mice (*R3^fl/fl^Cd8-Cre*) develop significantly faster tumor growth compared with control mice. Although Programmed death protein 1 (PD-1) blockade partially restores anti-tumor responses in these mice, the therapeutic effect remains markedly weaker than that observed in control animals. In human melanoma samples, RASAL3 expression is reduced in tumor-infiltrating CD8^+^ T cells. Furthermore, patients with metastatic melanoma show lower RASAL3 expression in primary tumors compared with patients without metastasis, and higher RASAL3 expression in CD8^+^ T cells within primary lesions is associated with improved overall survival. Together, these findings reveal that RASAL3 is required for the maintenance of CD8^+^ T cell survival and effector activity. Reduced RASAL3 expression may represent a mechanism by which tumors evade immune surveillance, and RASAL3 expression in CD8^+^ T cells may serve as a potential biomarker for predicting clinical outcomes in melanoma.

## Introduction

Ras signaling plays a pivotal role in a wide array of biological processes across nearly all cell types, including immune cells (1). Stimulation of Ras by exogenous signals typically triggers the activation of a downstream signaling cascade composed of rapidly accelerated fibrosarcoma (Raf), mitogen-activated protein kinase extracellular signal-regulated kinase kinase (MEK), and extracellular signal-regulated kinase (ERK) (2). This signaling axis ultimately activates transcription factors, such as nuclear factor kappa-light-chain-enhancer of activated B cells (NF-κB) and activator protein 1 (AP-1) (3, 4), leading to the enhanced expression of genes associated with cell survival, proliferation, and various effector functions (5, 6). In immune cells such as T cells, Ras signaling is driven by T cell receptor (TCR)-mediated activation, which increases the expression of crucial genes associated with their functions in forming adaptive immunity (7).

While Ras activation is essential, the precise regulation of this signaling is equally indispensable, as Ras hyperactivation frequently disrupts cellular homeostasis (8). For instance, it is well-documented that aberrant Ras hyperactivation triggers activation-induced cell death (AICD) or oncogene-induced senescence in healthy primary cells (9, 10). Conversely, in immortalized cells such as cancer, constitutive Ras activation contributes to rapid, unrestrained proliferation (11). Ras cycles between two conformational states: an “inactive” GDP-bound form (Ras-GDP) and an “active” GTP-bound form (Ras-GTP) (12). The conversion from the resting to the active state is mediated by guanine nucleotide exchange factors (GEFs), which facilitate the exchange of GDP for GTP (13). To terminate Ras signaling, Ras GTPase-activating proteins (RasGAPs) play a dominant role by accelerating the intrinsic hydrolytic activity of the Ras small GTPase, converting GTP back to GDP and thereby silencing the signal (14). Consequently, the loss of RasGAP function can be catastrophic for cellular regulation; indeed, the downregulation or mutation of RasGAPs is frequently identified in various malignancies (15, 16).

Due to the cell-type-specific nature of Ras signaling (17–19), identifying the predominant RasGAP within a specific lineage is critical to understanding how cellular activity is regulated. We previously identified Ras protein activator like 3 (RASAL3) as a RasGAP that functions predominantly within the hematopoietic lineage, specifically in natural killer T (NKT) cells and neutrophils (18, 19). In these populations, RASAL3 is essential for maintaining population size, differentiation, and overall function (18, 19). Subsequent study also identified an important role of RASAL3 in CD4^+^ T cells (20). However, the specific role of RASAL3 in CD8^+^ T cells remains poorly understood. Given that CD8^+^ T cells are central effectors of immune responses against diseases such as cancer (21), elucidating their regulatory mechanisms is vital for assessing and improving the efficacy of anti-tumor immunity.

In this study, we investigate the role of RASAL3 in the homeostasis and function of CD8+ T cells using both *in vitro* and *in vivo* murine B16-F10 melanoma models. Furthermore, we utilized human clinical samples to explore whether the downregulation of RASAL3 in CD8^+^ T cells can serve as a predictive biomarker for melanoma progression and distant metastasis.

## Materials and methods

### Tumor model

To establish a murine melanoma model, mice were inoculated with B16-F10 cells (5.0 × 10^5^ cells in 100 μL of PBS) via subcutaneous (s.c.) injection into their back skin, following a method described previously (22). For immune checkpoint blockade (ICB) treatment, mice received intraperitoneal (i.p.) injections of an isotype control antibody (Rat IgG) or an anti-PD-1 monoclonal antibody (mAb) (200 μg in 100 μL of PBS each) every 3 days. Antibody details are provided in **Supplementary Table 1**. Tumor-bearing mice were sacrificed at day 21 post-tumor inoculation (pti), and the tumors were subjected to immune analysis by flow cytometry. Alternatively, to obtain tumor antigen-specific CD8^+^ T cells (expressing TCRs reactive to SIINFEKL), B16-OVA cells (5.0 × 10^5^ cells in 100 μL of PBS) were inoculated into the back skin via s.c. injection. All animal experimental protocols were reviewed and approved by the animal ethics committees of the University of South China (Approval No. 2024-12) and Shibata Gakuen University (Approval No. 2018).

### *In vitro* CD8^+^ T cell stimulation and functional assay

For the Ras activation assay, CD8^+^ T cells (5.0 × 10^6^/mL) were cultured in complete RPMI medium (RPMI 1640 supplemented with 10% fetal bovine serum (FBS) and 1% penicillin-streptomycin) with or without anti-CD3/CD28 microbeads (Thermo Fisher Scientific, Waltham, MA, USA) at a cell:bead ratio of 1:10 at 37 °C for 5 min. Cells were immediately subjected to quantification of Ras-GTP levels using the Ras G-LISA GTPase Activation Assay Kit (Cytoskeleton, Denver, CO, USA) and measurement of pErk levels by flow cytometry. For the cell proliferation assay, CD8^+^ T cells (1.0 × 10^6^/mL) were first stained with carboxyfluorescein succinimidyl ester (CFSE) (Thermo Fisher Scientific), then cultured in complete RPMI medium with anti-CD3/CD28 microbeads (at a cell:bead ratio of 1:1) at 37 °C for 72 h. The frequency of proliferated cells was analyzed by flow cytometry following CFSE dilution every 12 h. At the end of the assay, the apoptotic status of the cells was analyzed via Annexin V/7-Aminoactinomycin D (AAD) staining using flow cytometry. Additionally, active caspase-3/7 levels were analyzed by flow cytometry in CD8^+^ T cells at baseline (immediately after isolation) and after stimulation with anti-CD3/CD28 microbeads (cell:bead ratio = 1:10) at 37 °C for 24 min using CellEvent™ Caspase-3/7 detection reagents (Thermo Fisher Scientific). For MEK inhibition, CD8^+^ T cells were pre-treated with vehicle (dimethyl sulfoxide; DMSO) or U0126 (10 μM) at 37 °C for 60 min prior to subsequent treatment.

### *In vitro* tumor killing assay

To obtain tumor antigen-specific CD8^+^ T cells from B16-OVA-inoculated mice, SIINFEKL-H-2K^b^-tetramer^+^ (SIINFEKL-Tet^+^) CD8^+^ T cells were isolated from the draining lymph nodes of tumor-bearing mice (day 14 pti) using a cell sorter. The isolated CD8^+^ T cells were expanded *in vitro* in the presence of SIINFEKL peptide (1 μg/mL) and IL-2 (10 ng/mL) for 5 days. B16-OVA cells were seeded in 24-well plates at 1.0 × 10^6^/mL in the presence of IFN-γ (10 ng/mL) and incubated at 37 °C overnight. CD8^+^ T cells were added to the B16-OVA culture at a target:effector ratio of 1:10 and incubated at 37 °C for 6 h. The B16-OVA cells were then harvested and stained with 7-AAD at room temperature (RT) for 15 min. The percentage of 7-AAD^+^ cells within the B16-OVA population (CD45^-^) was used to identify dead cells. The specific killing rate was calculated using the following formula: Specific killing (%) = [(Experimental 7-AAD^+^ (%) - Spontaneous 7-AAD^+^ (%))/(100 - Spontaneous 7-AAD^+^ (%))] × 100.

### Immunofluorescence staining of human melanoma tissue

Human tissue sections (adjacent healthy skin, n=18; melanoma lesions, n=112) were obtained from Xiangya Hospital (Hunan, China) and Shanghai Zhuoli Biotechnology Co., Ltd. (Shanghai, China). All samples were collected from patients who provided informed consent. Sample origin and patient information are detailed in **Supplementary Table 2**. Paraffin-embedded tissue specimens were deparaffinized in xylene, rehydrated through a graded ethanol series, subjected to antigen retrieval, and immersed in a 0.3% hydrogen peroxide solution. The slides were washed three times with PBS, then incubated with 5% bovine serum albumin (BSA) at RT for 30 min. The slides were then incubated with primary antibodies at 4 °C overnight, followed by incubation with fluorochrome-conjugated secondary antibodies at RT for 60 min. The slides were washed twice with PBS, mounted with ProLong™ Gold Antifade Mountant (Thermo Fisher Scientific), and coverslipped. The slides were analyzed using digital image analysis (DIA) techniques with the fully automated VIS DIA VisioMorph system (Visiopharm^®^, Hoersholm, Denmark). For data analysis, multi-component TIFF images were exported from inForm software (Akoya Biosciences, Menlo Park, CA, USA) and analyzed with Visiopharm software. The antibodies used for staining are listed in **Supplementary Table 1**. This study was approved by the Medical Ethics Committee of the University of South China (Approval No. 20240028).

### Statistics

Welch’s t-test, Mann-Whitney U test, one-way analysis of variance (ANOVA) and two-way ANOVA were used to analyze the data for significant differences. Values of *p* < 0.05, p < 0.01, and *p* < 0.001 were considered statistically significant.

## Results

### RASAL3 deficiency impairs CD8^+^ T cell survival upon TCR stimulation

We first investigated the role of RASAL3 in CD8^+^ T cell homeostasis using *in vitro* assays. CD8^+^ T cells were isolated from the spleens of wild-type (WT) and RASAL3-knockout (KO) mice (18, 19), hereafter referred to as WT and ΔR3 CD8^+^ T cells, respectively. These cells were subjected to *in vitro* assays evaluating Ras activation, proliferation, and apoptosis in the presence or absence of T cell receptor (TCR) stimulation using anti-CD3/CD28 microbeads (**Figure 1A**).

**Figure 1:**
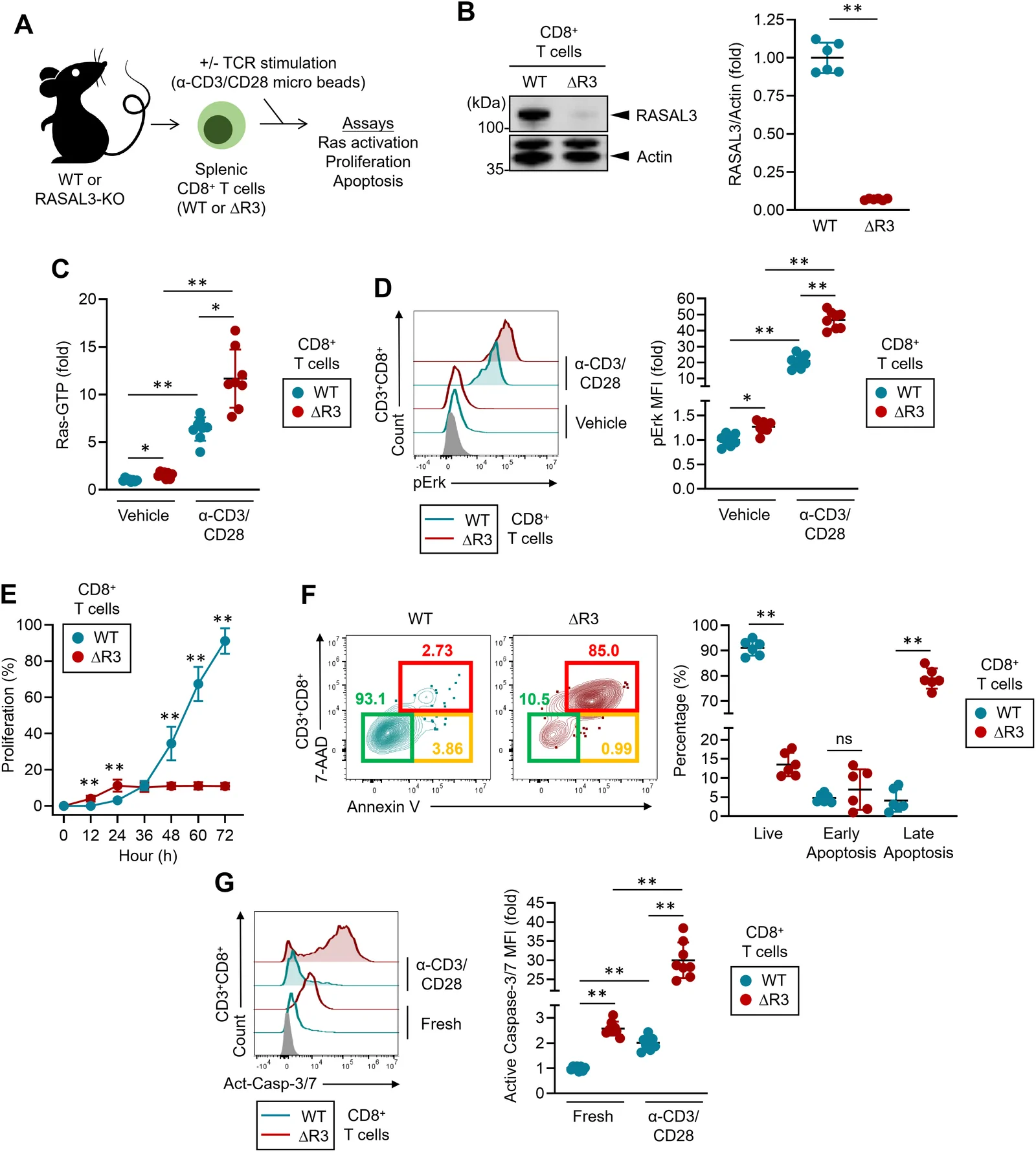
RASAL3 deficiency induces aggressive apoptosis in CD8^+^ T cells upon TCR stimulation, **(A)** Experimental design of the *in vitro* functional assays in CD8^+^ T cells. Splenic CD8^+^ T cells were isolated from WT or RASAL3-KO mice (referred to as WT or ΔR3 CD8^+^ T cells). CD8^+^ T cells were cultured *in vitro* with or without anti-CD3/CD28 microbead stimulation, and Ras activity, proliferation, and apoptotic status were subsequently analyzed in each culture. **(B)** RASAL3 expression in splenic WT or ΔR3 CD8^+^ T cells analyzed by WB. Representative WB images (left) and cumulative quantitative band intensities (right) of RASAL3 are shown. **(C)** Ras activation assay. CD8^+^ T cells were incubated with or without anti-CD3/CD28 microbeads at 37 °C for 5 min, and were immediately subjected to the quantification of Ras-GTP levels by a colorimetric assay. **(D)** Erk activation assay. CD8^+^ T cells were incubated following the protocol used in **(C)**. pErk levels were analyzed by flow cytometry. Representative histograms (left) and cumulative MFI values (fold change) (right) are shown. **(E)** Proliferation assay. CFSE-labeled CD8^+^ T cells were cultured with anti-CD3/CD28 microbeads at 37 °C, and the proliferation rate was analyzed every 12 h up to 72 h. The time-dependent proliferation rate is shown. **(F)** Apoptosis assay of CD8^+^ T cells. CD8^+^ T cells were incubated with anti-CD3/CD28 microbeads at 37 °C for 72 h, then subjected to flow cytometry to investigate apoptotic status following Annexin V/7-AAD staining. The populations were classified as live (Annexin V^–^7-AAD^–^), early apoptotic (Annexin V^+^7-AAD^–^), and late apoptotic (Annexin V^+^7-AAD^+^). Representative plots (left) and cumulative percentages of cellular status (right) are shown. **(G)** Expression of active caspase-3/7. Active caspase-3/7 levels were analyzed in either freshly isolated or stimulated (incubated with anti-CD3/CD28 microbeads at 37 °C for 24 h) CD8^+^ T cells by flow cytometry. Representative histograms (left) and cumulative MFI values (fold change) of active caspase-3/7 are shown. The cumulative data are shown as the mean ± standard deviation (SD) of six **(B, F)** to eight (others) samples from three independent experiments. All MFI fold-change values were calculated by setting the mean MFI value of the control group to 1. Welch’s t-test or one-way ANOVA was used to determine statistical significance. *p < 0.01 and **p < 0.001; ns, not significant.

Western blot analysis confirmed a >90% depletion of RASAL3 protein expression in ΔR3 CD8^+^ T cells compared to WT controls (**Figure 1B**). As RASAL3 functions as a RasGAP that negatively regulates Ras signaling (18–20), we next examined whether its deficiency alters Ras activation. Under basal conditions, Ras-GTP levels were mildly elevated (1.56-fold) in ΔR3 CD8^+^ T cells compared to WT cells. While TCR stimulation dramatically increased Ras-GTP levels in both populations, the response was significantly exacerbated in ΔR3 CD8^+^ T cells (11.7-fold upregulation relative to the WT basal level, compared to a 6.36-fold increase in WT cells) (**Figure 1C**). We subsequently quantified the levels of phosphorylated ERK (pERK), a downstream effector of Ras (2), using mean fluorescence intensity (MFI) via flow cytometry. Mirroring the Ras-GTP dynamics, basal pERK levels were elevated 1.27-fold in ΔR3 CD8^+^ T cells. Upon TCR stimulation, pERK expression surged drastically in ΔR3 CD8^+^ T cells (46.6-fold increase from WT basal), whereas WT cells exhibited a more moderate 20.9-fold increase (**Figure 1D**). The MEK inhibitor U0126 significantly reduced Erk hyperactivation in ΔR3 CD8^+^ T cells, which confirms that the elevated pErk observed in RASAL3-deficient CD8^+^ T cells is driven by hyperactive Ras signaling cascade (**Supplementary Figure 1A**) (23).

To assess the functional consequences of this Ras hyperactivation, we tracked cell proliferation via CFSE dilution following TCR stimulation (24). Within the first 24 hours, ΔR3 CD8^+^ T cells exhibited a rapid early proliferative response compared to WT cells (11.2 ± 3.28% proliferated). However, this initial burst was transient, and the frequency of proliferated ΔR3 CD8^+^ T cells plateaued, remaining at 11.0% by 72 hours. In contrast, while WT cells initiated proliferation more slowly, they exhibited a sustained, time-dependent expansion, reaching 91.2% proliferation by 72 hours (**Figure 1E**). Treatment with U0126 rescued the proliferation of ΔR3 CD8^+^ T cells (28.3 ± 3.02%); however, this rate remained significantly lower than that of MEK uninhibited WT cells (**Supplementary Figure 1B**). To determine the cause of this aborted proliferation in ΔR3 CD8^+^ T cells, and considering that Ras hyperactivation can trigger apoptosis (likely via AICD) (8), we evaluated cell viability. Flow cytometric analysis of Annexin V and 7-AAD staining at 72 hours (from the cultures in **Figure 1E**) revealed striking differences. While the vast majority of WT CD8^+^ T cells remained viable (91.1 ± 3.05% Annexin V^−^7-AAD^−^), the ΔR3 cultures exhibited massive cell death, with more than 85% of cells in early (6.98 ± 5.28% Annexin V^+^7-AAD^−^) or late (78.9 ± 4.00% Annexin V^+^7-AAD^+^) apoptosis (**Figure 1F**). Furthermore, U0126 treatment significantly decreased Annexin V levels in ΔR3 CD8^+^ T cells, indicating that suppressing tha hyperactivated Ras signaling cascade blunted the apoptotic cell death triggered by RASAL3 deficiency (**Supplementary Figure 1C**). Consistent with this, active caspase-3/7 levels were markedly elevated in ΔR3 CD8^+^ T cells under both basal (2.5-fold) and TCR-stimulated conditions (30-fold relative to WT basal) (**Figure 1G**).

These behaviors of RASAL3-deficient CD8^+^ T cells are similar to those of cells undergoing AICD, as described in previous reports (25, 26). Taken together, these findings demonstrate that RASAL3 deficiency drives the hyperactivation of Ras and its downstream signaling cascades, ultimately triggering apoptotic cell death in CD8^+^ T cells upon TCR stimulation.

### RASAL3 deficiency impairs the function of CD8^+^ T cells

Although TCR stimulation dramatically decreased the survival rate of ΔR3 CD8^+^ T cells, we found that a subset of cells remained viable even under stimulated conditions (**Figures 1E, F**). To understand the immunological contribution of these surviving ΔR3 CD8^+^ T cells, we assessed their function with anti-CD3/CD28 microbeads using isolated splenic CD8^+^ T cells (**Figure 2A**).

**Figure 2:**
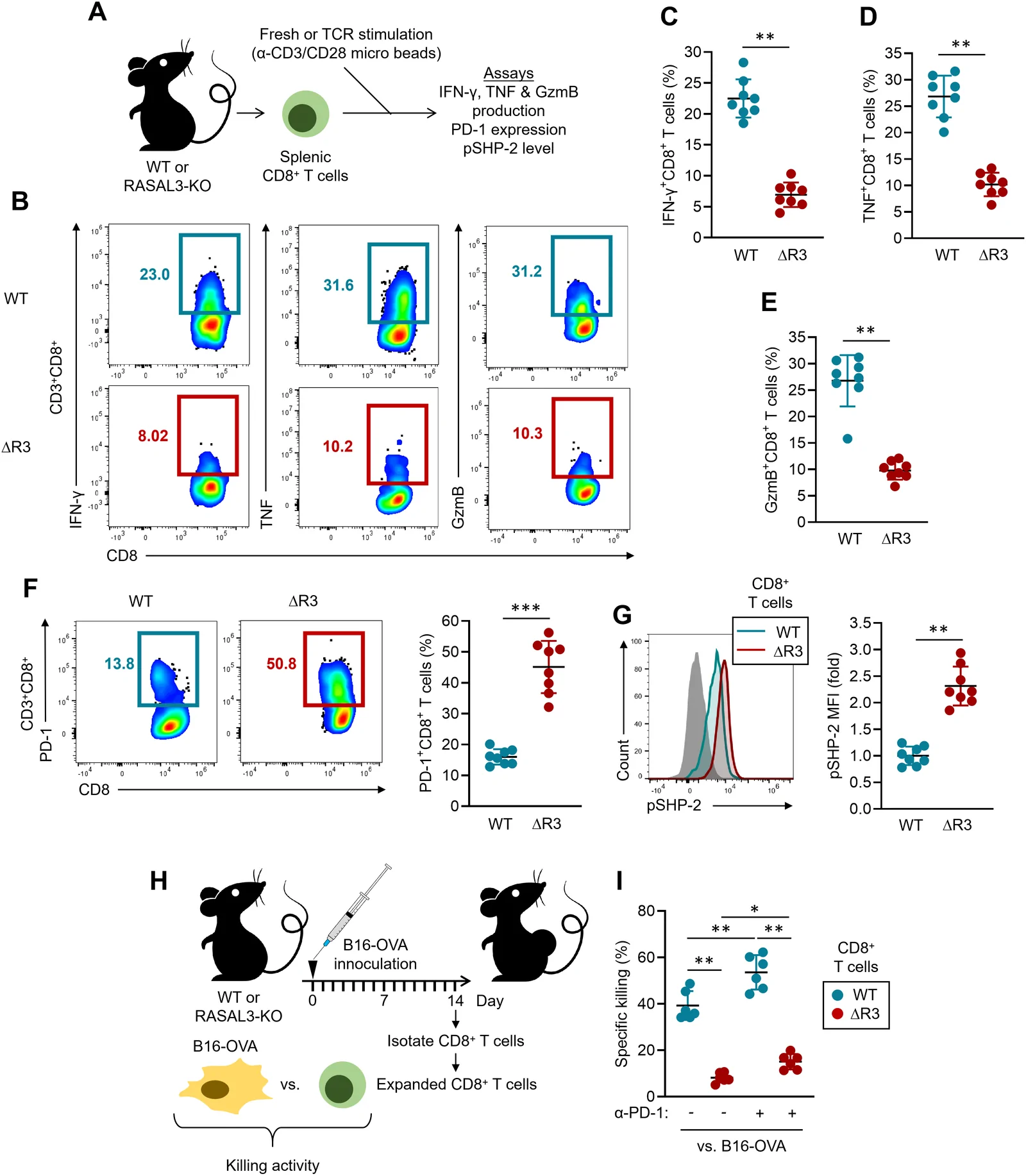
RASAL3 deficiency impairs the effector function of CD8^+^ T cells. **(A)** Experimental design of the *in vitro* functional assay of CD8^+^ T cells. Splenic CD8^+^ T cells were isolated from WT or RASAL3-KO mice (referred to as WT or ΔR3 CD8^+^ T cells) and stimulated with anti-CD3/CD28 microbeads at 37 °C for 72 h. Cytokine and GzmB production, as well as PD-1 and pSHP-2 levels, were analyzed by flow cytometry. **(B)** Representative plots of IFN-γ, TNF and GzmB production in CD8^+^ T cells, **(C-E)** Cumulative percentage values of IFN-γ^+^ **(C)**, TNF^+^ **(D)** and GzmB^+^ **(E)** CD8^+^ T cells. **(F)** Representative plots (left) and cumulative percentage values (right) of PD-1^+^CD8^+^ T cells. **(G)** Representative histograms (left) and cumulative MFI values (fold change) (right) of pSHP-2 in CD8^+^ T cells. **(H)** Experimental design of the *in vitro* tumor killing assay. Tumor antigen-specific (SIINFEKL-H-2K^b^-Tet^+^) CD8^+^ T cells were isolated from B16-OVA-inoculated mice at day 14 pti. The CD8^+^ T cells were expanded *in vitro* for 5 days, then co-cultured with B16-OVA cells (at a target:effector ratio of 1:10) in the presence of an isotype control antibody or anti-PD-1 mAb at 37 °C for 6 h. The percentage of dead B16-OVA cells was measured by flow cytometry following 7-AAD staining. **(I)** Cumulative percentage of specific killing of B16-OVA cells by CD8^+^ T cells. The cumulative data are shown as the mean ± standard deviation (SD) of six **(I)** to eight (others) samples from three independent experiments. All MFI fold-change values were calculated by setting the mean MFI value of the control group to 1. Welch’s t-test or one-way ANOVA was used to determine statistical significance. *p<0.05, **p<0.01 and ***p<0.001; ns, not significant.

Following stimulation, WT CD8^+^ T cells continued to express higher levels of these cytokines and GzmB than ΔR3 cells (WT vs. ΔR3: IFN-γ, 22.5 ± 3.10% vs. 6.93 ± 1.98%; TNF, 26.8 ± 3.95% vs. 10.2 ± 2.22%; and GzmB, 26.8 ± 4.85% vs. 9.78 ± 1.73%) (**Figures 2B–E**).

This functional downregulation in ΔR3 CD8^+^ T cells was associated with a dramatic upregulation of the immune checkpoint molecule programmed cell death protein 1 (PD-1) upon TCR stimulation (WT vs. ΔR3: 16.0 ± 2.51% vs. 45.1 ± 8.47%) (**Figure 2F**) (27). Other ICMs, such as lymphocyte-activation gene 3 (LAG-3), T-cell immunoglobulin and mucin-domain containing-3 (Tim-3), T cell immunoreceptor with Ig and ITIM domains (TIGIT), V-domain Ig suppressor of T cell activation (VISTA), Cytotoxic T-lymphocyte associated protein 4 (CTLA-4), CD160 and CD244, also showed upregulations in ΔR3 CD8^+^ T cells (**Supplementary Figure 2A**) (28). Additionally, we found that the level of phosphorylated Src homology region 2 domain-containing phosphatase-2 (pSHP-2) was also increased in ΔR3 CD8^+^ T cells (2.31-fold) (**Figure 2G**) (29). Furthermore, we identified that the thymocyte selection-associated high mobility group box (TOX) expression level was significantly increased in ΔR3 CD8^+^ T cells compared to WT cells upon TCR stimulation (**Supplementary Figure 2B**) (30). These signatures provide proof that RASAL3 deficiency causes CD8+ T cells to fall into an exhausted status.

We further investigated the influence of RASAL3 deficiency on the cytotoxic activity of CD8^+^ T cells against cancer cells. Tumor antigen-specific (SIINFEKL-Tet^+^) CD8^+^ T cells were isolated from the draining lymph nodes (dLNs) of B16-OVA-inoculated mice (WT or RASAL3-KO) and were expanded *ex vivo* in the presence of SIINFEKL peptide (the B16-OVA-expressed tumor antigen) and interleukin-2 (IL-2) for 5 days. The expanded CD8^+^ T cells were subjected to an *in vitro* killing assay against B16-OVA cells using a coculture system (target-to-effector ratio = 1:10). The percentage of killed B16-OVA cells was then assessed by flow cytometry following 7-AAD staining (**Figure 2H**). While WT CD8^+^ T cells killed 39.2% of the B16-OVA cells, ΔR3 CD8^+^ T cells killed only 8.14%. PD-1 blockade significantly improved the killing efficiency of WT CD8^+^ T cells against B16-OVA cells, increasing it to 53.6%. Although this immune checkpoint blockade (ICB) also improved the killing efficiency of ΔR3 CD8^+^ T cells, the rate remained drastically lower than that of WT cells, reaching only 15.1% (**Figure 2I**).

Thus, RASAL3 deficiency impairs the function of CD8^+^ T cells, even in the surviving cells that escape AICD caused by Ras hyperactivation. While PD-1 blockade helps improve the cytotoxic activity of RASAL3-deficient CD8^+^ T cells, its effect is limited, and the tumor killing rate remains far below that of WT cells.

### CD8^+^ T cell-specific RASAL3 deficiency fails to resist tumor progression

We investigated how CD8^+^ T cell-specific RASAL3 deficiency impairs anti-tumor immunity employing a B16-F10-derived melanoma mouse model. *Rasal3^flox/flox^* (*R3^fl/fl^*, control) or *Rasal3^flox/flox^Cd8-Cre* (*R3^fl/fl^Cd8-Cre*; CD8^+^ T cell-specific RASAL3-deficient) mice were inoculated with B16-F10 cells. The mice were administered either an isotype control antibody or an anti-PD-1 mAb on days 7, 10, 13, 16, and 19. Tumor growth was monitored until day 21 post-tumor inoculation (pti), and intratumoral CD8^+^ T cell function was analyzed at the end of the experiment (**Figure 3A**).

**Figure 3:**
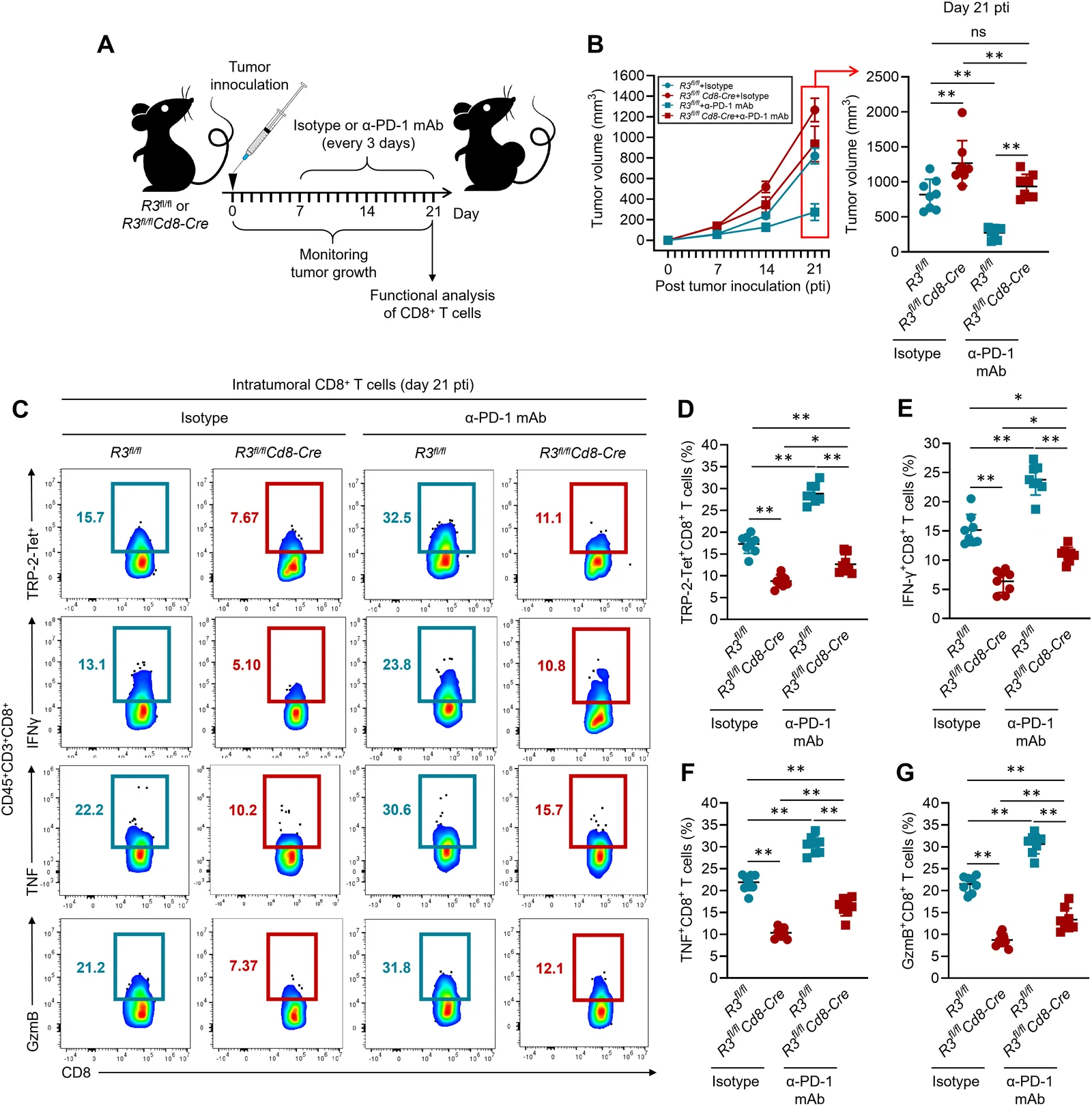
CD8^+^ T cell-specific RASAL3 deficiency impairs anti-tumor activity, resulting in tumor progression. **(A)** Experimental design of the murine syngeneic tumor model. *R3^fl/fl^* or *R3^fl/fl^ Cd8-Cre* mice were inoculated with B16-F10 on their back skin via s.c. injection. The mice were administered an isotype control or anti-PD-1 mAb via i.p. injection every 3 days starting from day 7 pti (days 7, 10, 13, 16, and 19 pti). Tumor growth was monitored throughout the experimental period until day 21 pti. The mice were sacrificed, and intratumoral CD8^+^ T cell function was characterized in each mouse at day 21 pti. **(B)** Tumor volumes. Time-dependent tumor progression (left) and cumulative tumor volume at day 21 pti (right) are shown. **(C)** Representative plots of tumor antigen-specific (TRP-2-Tet^+^) and effector CD8^+^ T cells in tumor microenvironment (TME). The frequencies of TRP-2-Tet^+,^IFN-γ^+^, TNF^+^, GzmB^+^CD8^+^ T cells were analyzed by flow cytometry. Cumulative percentage values of these subsets are shown in **(D–G)**. The cumulative data are shown as the mean ± standard deviation (SD) of eight samples from three independent experiments. Two-way ANOVA was used to determine statistical significance. *p < 0.01 and **p < 0.001; ns, not significant.

RASAL3 protein expression decreased by more than 90% in CD8^+^ T cells from *R3^fl/fl^Cd8-Cre* mice compared to control mice. However, RASAL3 expression was maintained in CD4^+^ T cells of *R3^fl/fl^Cd8-Cre* mice (**Supplementary Figure 3**). *R3^fl/fl^Cd8-Cre* mice showed normal thymic T cell development (**Supplementary Figure 4**). Additionally, the frequencies and numbers of immune cells were equivalent in the peripheral blood and spleen of *R3^fl/fl^* and *R3^fl/fl^Cd8-Cre* mice (**Supplementary Figure 5**). We found that tumor growth was accelerated in *R3^fl/fl^Cd8-Cre* mice compared to *R3^fl/fl^* controls. A significant increase in tumor volume was first identified in *R3^fl/fl^Cd8-Cre* mice on day 14 pti compared to control mice, ultimately resulting in a 30% increase in tumor volume due to CD8^+^ T cell-specific RASAL3 deficiency in day 21 pti (*R3^fl/fl^*: 818.7 ± 217.7 mm^3^ vs. *R3^fl/fl^Cd8-Cre*: 1266.5 ± 305.8 mm^3^). PD-1 blockade suppressed tumor progression in both control and *R3^fl/fl^Cd8-Cre* mice. However, its effect was more remarkable in control mice than in *R3^fl/fl^Cd8-Cre* mice (*R3^fl/fl^*: -62.9 ± 18.1% vs. *R3^fl/fl^Cd8-Cre*: -23.5 ± 18.1% tumor volume reduction) (**Figure 3B**; **Supplementary Figure 6**). Intratumoral CD8^+^ T cell function was evaluated by measuring the frequency of TRP-2-H-2K^b^ tetramer^+^ (named TRP-2-Tet^+^) cells (tumor antigen-specific) alongside the expression of IFN-γ, TNF and GzmB (22, 31). RASAL3 deficiency significantly decreased the frequency of TRP-2-Tet^+^ cells within the intratumoral CD8^+^ T cell population (*R3^fl/fl^*: 17.3 ± 2.11% vs. *R3^fl/fl^Cd8-Cre*: 8.79 ± 1.46%). While PD-1 blockade increased this frequency in both strains during the tumor challenge, the efficacy was superior in *R3^fl/fl^* mice, leading to a 28.8% expanded population compared to only 12.7% in *R3^fl/fl^Cd8-Cre* mice (**Figures 3C, D**). Cytokines and GzmB expression levels were also significantly decreased by RASAL3 deficiency in intratumoral CD8^+^ T cells compared to controls (*R3^fl/fl^* vs. *R3^fl/fl^Cd8-Cre*: IFN-γ^+^, 15.2 ± 2.65% vs. 6.37 ± 1.90%; TNF^+^, 21.9 ± 1.90% vs. 10.3 ± 1.23%; and GzmB^+^, 21.5 ± 1.87% vs. 8.73 ± 1.49%). Again, PD-1 blockade partially restored these effector molecules; however, its efficacy was far better in *R3^fl/fl^* mice than in *R3^fl/fl^Cd8-Cre* mice (**Figures 3C, E–G**). Of note, the functions of antigen-presenting cells (APCs), such as macrophages and dendritic cells (DCs), were equivalent between *R3^fl/fl^* and *R3^fl/fl^Cd8-Cre* mice. This confirms that the functional downregulation was directly driven by the loss of RASAL3 intrinsically within CD8^+^ T cells during the tumor challenge (**Supplementary Figure 7**). Collectively, these data demonstrate that CD8^+^ T cell-specific RASAL3 deficiency impairs anti-tumor immunity, thereby accelerating tumor progression.

### RASAL3 downregulation in lesional CD8^+^ T cells is a signature of increased metastasis in melanoma patients

Given our findings in the mouse model, we investigated the clinical relevance of RASAL3 expression in CD8^+^ T cells and its association with melanoma progression in human samples. We examined primary human melanoma tissues (n=112) and adjacent healthy skin controls (n=18, obtained from a subset of these patients) (**Supplementary Table 2**) using multicolor immunofluorescence staining. Tissue sections were stained for RASAL3 and CD8, and the expression levels of these proteins were quantified using digital image analysis (DIA) techniques (32) (**Figure 4A**). We found that RASAL3 expression strongly colocalized with CD8^+^ T cells in both healthy and melanoma tissues, as confirmed by Spearman’s correlation analysis (*r* = 0.8314, *p* < 0.0001) (**Figures 4B, C**; **Supplementary Figure 8**). CD8 signals were significantly increased in melanoma lesions compared to adjacent healthy skin, implying enhanced infiltration of CD8^+^ T cells into the tumors (**Figure 4D**). Conversely, RASAL3 expression was significantly decreased in melanoma lesions compared to control tissues (**Figure 4E**). Under microscopic analysis, we observed that RASAL3 expression intensities varied widely across different melanoma tissues (**Supplementary Figure 8**). Interestingly, the expression intensities of RASAL3 and CD8 did not exhibit a significant correlation within the melanoma tissues, suggesting that the intratumoral CD8^+^ T cell population is functionally heterogeneous and characterized by variable RASAL3 expression levels (**Figure 4F**). Notably, we found that RASAL3 expression was significantly lower in primary melanoma tissues originating from patients who had already developed distant metastasis at the time of biopsy compared to non-metastatic cases (n=17 vs. n=95) (**Figure 4G**). Because CD8 expression in the primary melanoma was comparable between patients with and without metastasis, the observed RASAL3 downregulation results from decreased RASAL3 expression in tumor-infiltrating CD8^+^ T cells in metastatic patients (**Supplementary Figure 9**). Furthermore, among the patients with metastatic disease (n=17), RASAL3 expression levels in the primary tumor positively correlated with overall survival duration following the initial diagnosis (**Figure 4H**).

**Figure 4:**
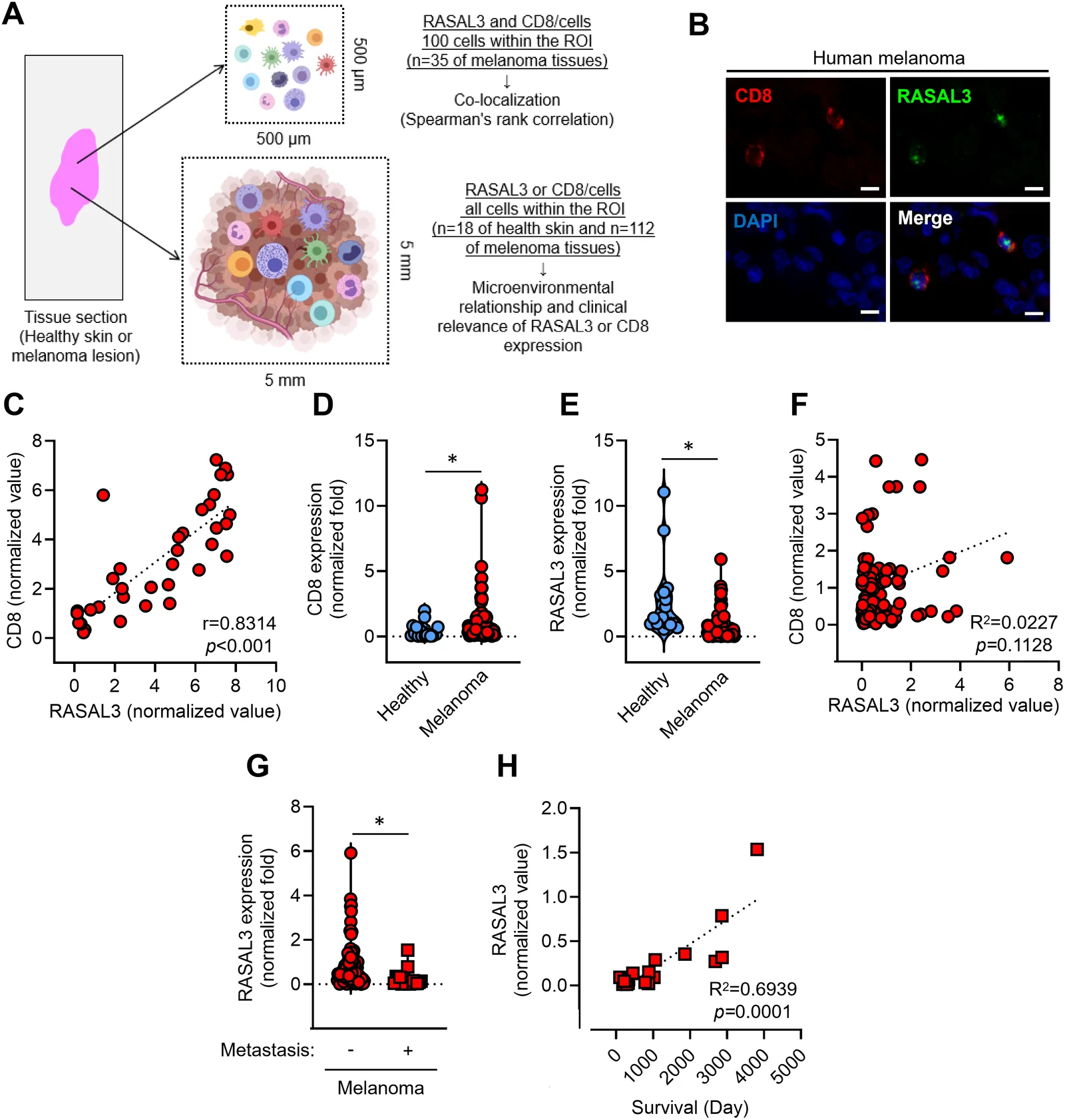
RASAL3 downregulation in lesional CD8^+^ T cells is a signature of melanoma progression. **(A)** Analysis strategy of human tissue samples. Adjacent healthy skin (n=18) and melanoma tissue (n=112) were subjected to immunofluorescence staining for RASAL3 and CD8. To investigate the colocalization of RASAL3 and CD8 signals within the cells, a total of 100 cells were randomly selected within a region of interest (ROI; 500 μm x 500 μm), and mean values for RASAL3 and CD8 were obtained (n=35 for melanoma tissues). These values were used for Spearman correlation. To investigate the relationship between CD8 or RASAL3 expression and clinical aspects, CD8 and RASAL3 expression intensities were detected in all cells within the ROI (5 mm x 5 mm) and mean values were used (healthy skin, n=18; melanoma tissues, n=112). **(B)** Representative immunofluorescence image of CD8 and RASAL3 colocalization in melanoma tissue. Scale bar = 10 μm. **(C)** Spearman correlation between CD8 and RASAL3 expression in melanoma tissues (n=35). D-E) CD8 **(D)** and RASAL3 **(E)** expression in healthy skin (n=18) and melanoma tissues (n=112), respectively. **(F)** Linear regression analysis of CD8 and RASAL3 expression in melanoma tissues (n=112). **(G)** RASAL3 expression in the original melanoma tissues derived from patients with (n=17) or without (n=95) metastasis. **(H)** Linear regression analysis of RASAL3 expression (original melanoma tissue) and survival length (after initial diagnosis for melanoma) in patients with metastasis (n=17). The cumulative data are shown as the mean ± standard deviation (SD) of all analyzed samples. Spearman correlation and linear regression were analyzed using GraphPad Prism 10. Mann-Whitney U test was used to determine statistical significance. *p < 0.05.

These results imply that RASAL3 downregulation in tumor-infiltrating CD8^+^ T cells correlates with, and potentially aggravates, tumor progression and metastasis in melanoma patients.

## Discussion

In this study, we demonstrate that RASAL3 plays a crucial role in the survival and function of CD8^+^ T cells, specifically under stimulated conditions. This newly identified immunobiological function of RASAL3 expands our understanding of the role of RasGAPs in immunity. Recent studies have actively highlighted the key roles of RasGAPs in T cell function, and members of the RASAL and RASA family have been shown to be essential for maintaining T cell differentiation and activity (33–36). In light of these trends, our study is highly timely, providing pivotal evidence of the critical role that RASAL3 plays in CD8^+^ T cell functionality. Consistent with previous findings, RASAL3 deficiency accelerated Ras signaling, resulting in the accumulation of Ras-GTP (18, 19). This hyperactivated state was prominently observed in stimulated CD8^+^ T cells, whereas it remained minimal under basal conditions. This hyperactivation triggered notable apoptotic cell death in RASAL3-deficient CD8^+^ T cells. Our results suggest that the caspase-3/7-mediated pathway plays a crucial role in the cell death of RASAL3-deficient CD8^+^ T cells following TCR stimulation. However, other mechanisms, such as metabolic defects frequently identified in tumor-infiltrating immune cells, may also contribute to this cell death in RASAL3-deficient CD8^+^ T cells (37, 38). We noted that a population of CD8^+^ T cells managed to survive despite the overactivation of Ras signaling induced by RASAL3 deficiency. These surviving RASAL3-deficient CD8^+^ T cells exhibited significantly reduced activity, accompanied by a marked upregulation of PD-1 compared to WT cells. While the precise mechanism allowing this specific subpopulation of RASAL3-deficient CD8^+^ T cells to survive remains to be elucidated, immune checkpoint molecule-associated functional regulation may serve as a compensatory mechanism promoting cell survival. Regarding PD-1 upregulation in surviving RASAL3-deficient CD8^+^ T cells, nuclear factor of activated T-cells, cytoplasmic 1 (NFATc1) may be involved as a master transcription factor driving its expression (39). Further studies are required to address this question by depleting or inhibiting NFATc1 in RASAL3-deficient CD8^+^ T cells.

To rescue the downregulated CD8^+^ T cell function induced by RASAL3 deficiency, we evaluated the efficacy of immune checkpoint blockade in a tumor model. PD-1 blockade significantly restored CD8^+^ T cell function in CD8^+^ T cell-specific RASAL3-deficient mice (*R3^fl/fl^ Cd8-Cre*), leading to notable tumor shrinkage compared to controls. However, neither CD8^+^ T cell function nor tumor volume reached levels equivalent to those observed in control mice (*R3^fl/fl^*). This outcome implies that PD-1 blockade alone is insufficient to fully rescue CD8^+^ T cells compromised by RASAL3 deficiency. While the finding was based on *in vitro* culture, we have also identified that the expression of other ICMs were upregulated by RASAL3 deficiency in CD8^+^ T cells upon TCR stimulation. This evidence implies that a combined ICB strategy targeting PD-1 and another ICM may contribute to restoring the function of RASAL3-deficient CD8^+^ T cells more effectively than targeting PD-1 alone (40, 41).

We concluded that the functional downregulation observed in RASAL3-deficient CD8^+^ T cells is intrinsic to the T cells themselves, as APC function was equivalent between R3^fl/fl^ and R3*^fl/fl^ Cd8-Cre* mice. However, the complexity of the interaction between CD8^+^ T cells and macrophages warrants further investigation in future studies (42).

We acknowledge that the near-complete depletion of RASAL3 in CD8^+^ T cells in our experimental system is an artificial, as it has not been reported under normal physiological conditions. However, the histological analysis of human melanoma samples provides us with interesting evidence. We identified a significant downregulation of RASAL3 in CD8^+^ T cells infiltrating human melanoma tissues compared to those in adjacent healthy skin. Interestingly, this downregulation of RASAL3 in CD8^+^ T cells positively correlated with the risk of metastasis in melanoma patients. This clinical observation aligns with our *in vivo* findings, where decreased RASAL3 expression impaired CD8^+^ T cell function and drove tumor progression in the B16-F10 model. Although we could not directly investigate Ras hyperactivation and apoptotic status in CD8^+^ T cells infiltrating human melanoma tissues, we hypothesize that the same molecular mechanism operates in human CD8^+^ T cells within the tumor microenvironment. Inducing the downregulation of RASAL3 in CD8^+^ T cells may represent a survival strategy employed by cancer cells to escape immune surveillance. Uncovering the precise mechanism driving RASAL3 downregulation in CD8^+^ T cells remains a critical objective for future studies. While these findings were not observed in immune cells, previous studies have shown that epigenetic modifications can downregulate RasGAPs, leading to hyperactivation of Ras signaling (43–45). Addressing this possibility is essential to elucidate the mechanism underlying RASAL3 downregulation in tumor-infiltrating CD8^+^ T cells.

In summary, our findings contribute significantly to the understanding of the immunobiological role of RASAL3 in CD8^+^ T cells, establishing its indispensable function in maintaining cellular homeostasis and T cell functionality. Specifically, this study provides the first report demonstrating that RASAL3 is a crucial functional regulator in CD8^+^ T cells. We have revealed the importance of RASAL3 as a key RasGAP in CD8^+^ T cell function using both murine and human models. While our current evidence is centered on anti-tumor immunity, we hypothesize that this crucial role of RASAL3 extends beyond oncology and may apply to other T cell lineages. Given our observations in human melanoma tissues, evaluating RASAL3 downregulation in CD8^+^ T cells holds promise as a biomarker for assessing the risk of functional decline in anti-tumor immunity and metastasis. Furthermore, co-evaluating the expression levels of immune checkpoint molecules, such as PD-1, could provide a more robust and comprehensive assessment of CD8^+^ T cell functional status.

## Data Availability

The original contributions presented in the study are included in the article/**Supplementary Material**. Further inquiries can be directed to the corresponding author.

## References

[1] Punekar SR Velcheti V Neel BG Wong KK . The current state of the art and future trends in RAS-targeted cancer therapies. Nat Rev Clin Oncol. (2022) 19:637–55. doi: 10.1038/s41571-022-00671-9 36028717 PMC9412785

[2] Downward J . Targeting RAS signalling pathways in cancer therapy. Nat Rev Cancer. (2003) 3:11–22. doi: 10.1038/nrc969 12509763

[3] Daniels MA Teixeiro E . The NF-κB signaling network in the life of T cells. Front Immunol. (2025) 16:1559494. doi: 10.3389/fimmu.2025.1559494 40370445 PMC12075310

[4] Kang SM Beverly B Tran AC Brorson K Schwartz RH Lenardo MJ . Transactivation by AP-1 is a molecular target of T cell clonal anergy. Science. (1992) 257:1134–8. doi: 10.1126/science.257.5073.1134 1509265

[5] Guo Q Jin Y Chen X Ye X Shen X Lin M . NF-κB in biology and targeted therapy: new insights and translational implications. Signal Transduct Target Ther. (2024) 9:53. doi: 10.1038/s41392-024-01757-9 38433280 PMC10910037

[6] Mao H Zhao X Sun SC . NF-κB in inflammation and cancer. Cell Mol Immunol. (2025) 22:811–39. doi: 10.1038/s41423-025-01310-w 40562870 PMC12310982

[7] Iborra S Soto M Stark-Aroeira L Castellano E Alarcón B Alonso C . H-ras and N-ras are dispensable for T-cell development and activation but critical for protective Th1 immunity. Blood. (2011) 117:5102–11. doi: 10.1182/blood-2010-10-315770 21444916

[8] Tanimoto Y Kizaki H . Proteasome inhibitors block Ras/ERK signaling pathway resulting in the downregulation of Fas ligand expression during activation-induced cell death in T cells. J Biochem. (2002) 131:319–26. doi: 10.1093/oxfordjournals.jbchem.a003106 11872160

[9] Feig LA Buchsbaum RJ . Cell signaling: life or death decisions of ras proteins. Curr Biol. (2002) 12:R259–61. doi: 10.1016/s0960-9822(02)00787-x 11937045

[10] Serrano M Lin AW McCurrach ME Beach D Lowe SW . Oncogenic ras provokes premature cell senescence associated with accumulation of p53 and p16INK4a. Cell. (1997) 88:593–602. doi: 10.1016/s0092-8674(00)81902-9 9054499

[11] Bahar ME Kim HJ Kim DR . Targeting the RAS/RAF/MAPK pathway for cancer therapy: from mechanism to clinical studies. Signal Transduct Target Ther. (2023) 8:455. doi: 10.1038/s41392-023-01705-z 38105263 PMC10725898

[12] Scheffzek K Ahmadian MR Kabsch W Wiesmüller L Lautwein A Schmitz F . The Ras-RasGAP complex: structural basis for GTPase activation and its loss in oncogenic Ras mutants. Science. (1997) 277:333–8. doi: 10.1126/science.277.5324.333 9219684

[13] Jun JE Rubio I Roose JP . Regulation of ras exchange factors and cellular localization of ras activation by lipid messengers in T cells. Front Immunol. (2013) 4:239. doi: 10.3389/fimmu.2013.00239 24027568 PMC3762125

[14] Scheffzek K Shivalingaiah G . Ras-specific GTPase-activating proteins-structures, mechanisms, and interactions. Cold Spring Harb Perspect Med. (2019) 9:a031500. doi: 10.1101/cshperspect.a031500 30104198 PMC6396337

[15] Grewal T Koese M Tebar F Enrich C . Differential regulation of rasGAPs in cancer. Genes Cancer. (2011) 2:288–97. doi: 10.1177/1947601911407330 21779499 PMC3128632

[16] McLaughlin SK Olsen SN Dake B De Raedt T Lim E Bronson RT . The RasGAP gene, RASAL2, is a tumor and metastasis suppressor. Cancer Cell. (2013) 24:365–78. doi: 10.1016/j.ccr.2013.08.004 24029233 PMC3822334

[17] Vigil D Cherfils J Rossman KL Der CJ . Ras superfamily GEFs and GAPs: validated and tractable targets for cancer therapy? Nat Rev Cancer. (2010) 10:842–57. doi: 10.1038/nrc2960 21102635 PMC3124093

[18] Saito S Kawamura T Higuchi M Kobayashi T Yoshita-Takahashi M Yamazaki M . RASAL3, a novel hematopoietic RasGAP protein, regulates the number and functions of NKT cells. Eur J Immunol. (2015) 45:1512–23. doi: 10.1002/eji.201444977 25652366

[19] Saito S Cao DY Victor AR Peng Z Wu HY Okwan-Duodu D . RASAL3 is a putative rasGAP modulating inflammatory response by neutrophils. Front Immunol. (2021) 12:744300. doi: 10.3389/fimmu.2021.744300 34777356 PMC8579101

[20] Muro R Nitta T Okada T Ideta H Tsubata T Suzuki H . The Ras GTPase-activating protein Rasal3 supports survival of naive T cells. PloS One. (2015) 10:e0119898. doi: 10.1371/journal.pone.0119898 25793935 PMC4368693

[21] Raskov H Orhan A Christensen JP Gögenur I . Cytotoxic CD8^+^ T cells in cancer and cancer immunotherapy. Br J Cancer. (2020) 124:359–67. doi: 10.1038/s41416-020-01048-4 32929195 PMC7853123

[22] Saito S Cao DY Bernstein EA Shibata T Jones AE Rios A . Peroxisome proliferator-activated receptor alpha is an essential factor in enhanced macrophage immune function induced by angiotensin-converting enzyme. Cell Mol Immunol. (2025) 22:243–59. doi: 10.1038/s41423-025-01257-y 39910334 PMC11868401

[23] You Y Niu Y Zhang J Huang S Ding P Sun F . U0126: Not only a MAPK kinase inhibitor. Front Pharmacol. (2022) 13:927083. doi: 10.3389/fphar.2022.927083 36091807 PMC9452634

[24] Saito S Kakizaki N Okuno A Maekawa T Tsuji NM . *Lactococcus lactis* subsp. *Cremoris* C60 restores T Cell Population in Small Intestinal Lamina Propria in Aged Interleukin-18 Deficient Mice. Nutrients. (2020) 12:3287. doi: 10.3390/nu12113287 33121026 PMC7693701

[25] Cheng LE Ohlén C Nelson BH Greenberg PD . Enhanced signaling through the IL-2 receptor in CD8+ T cells regulated by antigen recognition results in preferential proliferation and expansion of responding CD8+ T cells rather than promotion of cell death. Proc Natl Acad Sci USA. (2002) 99:3001–6. doi: 10.1073/pnas.052676899 11867736 PMC122462

[26] Zangari B Tsuji T Matsuzaki J Mohammadpour H Eppolito C Battaglia S . Tcf-1 protects anti-tumor TCR-engineered CD8+ T-cells from GzmB mediated self-destruction. Cancer Immunol Immunother. (2022) 71:2881–98. doi: 10.1007/s00262-022-03197-2 35460379 PMC9588092

[27] He X Xu C . PD-1: A driver or passenger of T cell exhaustion? Mol Cell. (2020) 77:930–1. doi: 10.1016/j.molcel.2020.02.013 32142689

[28] Saito S Bozorgmehr N Sligl W Osman M Elahi S . The role of coinhibitory receptors in B cell dysregulation in SARS-coV-2-infected individuals with severe disease. J Immunol. (2024) 212:1540–52. doi: 10.4049/jimmunol.2300783 38517295 PMC11075007

[29] Marasco M Berteotti A Weyershaeuser J Thorausch N Sikorska J Krausze J . Molecular mechanism of SHP2 activation by PD-1 stimulation. Sci Adv. (2020) 6:eaay4458. doi: 10.1126/sciadv.aay4458 32064351 PMC6994217

[30] Niu H Wang H . TOX regulates T lymphocytes differentiation and its function in tumor. Front Immunol. (2023) 14:990419. doi: 10.3389/fimmu.2023.990419 36969216 PMC10035881

[31] Peng Z Saito S . Creatine supplementation enhances anti-tumor immunity by promoting adenosine triphosphate production in macrophages. Front Immunol. (2023) 14:1176956. doi: 10.3389/fimmu.2023.1176956 37662917 PMC10471797

[32] Lin Z Yang H Kong Q Li J Lee SM Gao B . USP22 antagonizes p53 transcriptional activation by deubiquitinating Sirt1 to suppress cell apoptosis and is required for mouse embryonic development. Mol Cell. (2012) 46:484–94. doi: 10.1016/j.molcel.2012.03.024 22542455

[33] Thaker YR Raab M Strebhardt K Rudd CE . GTPase-activating protein Rasal1 associates with ZAP-70 of the TCR and negatively regulates T-cell tumor immunity. Nat Commun. (2019) 10:4804. doi: 10.1038/s41467-019-12544-4 31641113 PMC6805919

[34] Carnevale J Shifrut E Kale N Nyberg WA Blaeschke F Chen YY . RASA2 ablation in T cells boosts antigen sensitivity and long-term function. Nature. (2022) 609:174–82. doi: 10.1038/s41586-022-05126-w 36002574 PMC9433322

[35] Johansen KH Golec DP Okkenhaug K Schwartzberg PL . Mind the GAP: RASA2 and RASA3 GTPase-activating proteins as gatekeepers of T cell activation and adhesion. Trends Immunol. (2023) 44:917–31. doi: 10.1016/j.it.2023.09.002 37858490 PMC10621891

[36] Johansen KH Golec DP Huang B Park C Thomsen JH Preite S . A CRISPR screen targeting PI3K effectors identifies RASA3 as a negative regulator of LFA-1-mediated adhesion in T cells. Sci Signal. (2022) 15:eabl9169. doi: 10.1126/scisignal.abl9169 35857633 PMC9637254

[37] Drijvers JM Gillis JE Muijlwijk T Nguyen TH Gaudiano EF Harris IS . Pharmacologic screening identifies metabolic vulnerabilities of CD8^+^ T cells. Cancer Immunol Res. (2021) 9:184–99. doi: 10.1158/2326-6066.CIR-20-0384 33277233 PMC7864883

[38] Dai Z Liu J Shi K Peng X Jin Z Zheng R . Targeting ferroptosis in cancer therapy: Mechanisms, strategies, and clinical applications. Cell Investig. (2025) 1:100049. doi: 10.1016/j.clnves.2025.10004938826717

[39] Oestreich KJ Yoon H Ahmed R Boss JM . NFATc1 regulates PD-1 expression upon T cell activation. J Immunol. (2008) 181:4832–9. doi: 10.4049/jimmunol.181.7.4832 18802087 PMC2645436

[40] Patel SA Minn AJ . Combination cancer therapy with immune checkpoint blockade: mechanisms and strategies. Immunity. (2018) 48:417–33. doi: 10.1016/j.immuni.2018.03.007 29562193 PMC6948191

[41] Liu Y Yadav UP Chen C Wang P . Dual immune checkpoint blockade strategies for cancer therapy. Cell Investig. (2026) 2:100064. doi: 10.1016/j.clnves.2026.10006438826717

[42] Song Q Chaudagar K Zhang X Waller EK . T cell-macrophage crosstalk in GVHD and cancer immunotherapy. Cell Investig. (2025) 1:100035. doi: 10.1016/j.clnves.2025.100035 42273343 PMC13249391

[43] Jin H Wang X Ying J Wong AH Cui Y Srivastava G . Epigenetic silencing of a Ca(2+)-regulated Ras GTPase-activating protein RASAL defines a new mechanism of Ras activation in human cancers. Proc Natl Acad Sci USA. (2007) 104:12353–8. doi: 10.1073/pnas.0700153104 17640920 PMC1941473

[44] Li L Fan Y Huang X Luo J Zhong L Shu XS . Tumor suppression of ras GTPase-activating protein RASA5 through antagonizing ras signaling perturbation in carcinomas. iScience. (2019) 21:1–18. doi: 10.1016/j.isci.2019.10.007 31654850 PMC6820368

[45] McLaughlin SK Olsen SN Dake B De Raedt T Lim E Bronson RT . The RasGAP gene, RASAL2, is a tumor and metastasis suppressor. Cancer Cell. (2013) 24:365–78. doi: 10.1016/j.ccr.2013.08.004 24029233 PMC3822334

