# Supplementary material for: Ras protein activator-like 3 is a key Ras GTPase-activating protein that protects the survival and anti-tumor activity of CD8+ T cells
Source: Front Immunol. 2026 Sep 16;17:1893111. doi: 10.3389/fimmu.2026.1893111 (PMC13623836; doi:10.3389/fimmu.2026.1893111)
Supplement: Supplementary file 1 [file DataSheet1.pdf]

Supplementary Material

Ras protein activator-like 3 (RASAL3) is a key Ras GTPase-activating protein that protects the survival and anti-tumor activity of CD8$^+$ T cells

Zhenzi Peng, Xinyi Zhou, Alato Okuno, Derick Okwan-Duodu and Suguru Saito

Corresponding author
Suguru Saito, Ph.D.
E-mail: Suguru.Saito@cshs.org
TEL: +1-213-823-0661

---

1. Supplementary Protocols

Mice
C57BL/6 mice were purchased from Hunan SJA Laboratory Animal Co., Ltd (Hunan, China), and Japan SLC, Inc. (Shizuoka, Japan). CD8-Cre mice (C57BL/6-Tg(Cd8a-cre)1Itan/J) were purchased from The Jackson Laboratory (Bar Harbor, ME, USA). RASAL3-flox/flox (*R3*$^{fl/fl}$) and RASAL3-KO mice (global depletion of RASAL3) were generated following methods described in previous publications [18, 19]. *R3*$^{fl/fl}$ mice were crossed with CD8-Cre mice to generate CD8$^+$ T cell-specific RASAL3-depleted mice (*R3*$^{fl/fl}$*Cd8-Cre*). RASAL3-KO mice and *R3*$^{fl/fl}$*Cd8-Cre* mice were identified by genotyping PCR following methods described in previous reports [18, 19]. The primer sequences for genotyping PCR are shown in Supplemental Table 3. Depletion of RASAL3 in CD8$^+$ T cells was confirmed in all *R3*$^{fl/fl}$*Cd8-Cre* mice by flow cytometry before being used in the experiments.

Cell Isolation
To investigate the immune function of the tumor microenvironment (TME), cells were isolated from B16-F10-originated tumors. The tumors were excised, briefly washed in ice-cold PBS, and then minced with scissors in complete RPMI medium. The samples were further mechanically crushed through a 70 µm cell strainer, and the cells were pelleted by centrifugation at 300 × g for 5 min. The isolated cells were resuspended in complete RPMI medium and directly subjected to flow cytometry analysis. Splenocytes were isolated from spleens by mechanical crushing through a 70 µm cell strainer in complete RPMI medium. The cells were pelleted by centrifugation at 300 × g for 5 min and then incubated with 1X red blood cell lysis buffer at room temperature (RT) for 10 min, followed by washing with complete RPMI medium. The cells were pelleted again by centrifugation at 300 × g for 5 min. These isolated cells were used as splenocytes. CD8$^+$ T cells were isolated from splenocytes using a mouse CD8$^+$ T Cell Isolation Kit (Miltenyi Biotec, Bergisch Gladbach, Germany). All isolation procedures followed the manufacturer's instructions. The purity of the isolated CD8$^+$ T cells was assessed by flow cytometry, and samples with >90% purity were used for experiments.

Flow Cytometry
Flow cytometry analysis was performed using an LSR II or FACSAria I flow cytometer (BD Biosciences, Franklin Lakes, NJ, USA). For extracellular marker staining, samples were stained with fluorochrome-conjugated monoclonal antibodies (mAbs) or tetramers (tet) in the presence of an anti-CD16/CD32 mAb to block Fc gamma receptors (FcγRII/III) at 4°C for 30 min. Intracellular staining was performed using the BD Cytofix/Cytoperm™ Fixation/Permeabilization Kit (BD Biosciences). For intracellular staining of RASAL3, cytokines, or granzyme B, the extracellular-stained cells were fixed at

4°C for 20 min, followed by staining of the intracellular targets at 4°C for 30 min. For cytokine staining, samples were stimulated with PMA (100 ng/mL, Thermo Fisher Scientific) and ionomycin (250 ng/mL, Thermo Fisher Scientific) in the presence of GolgiStop™ (1 µg/mL, BD Biosciences) at 37°C for 6 h prior to intracellular staining. The antibodies used for flow cytometry analysis are listed in Supplemental Table 1. Data were analyzed using FlowJo™ 10 software (BD Biosciences).

Western Blotting (WB)
For protein isolation, cells were treated with RIPA buffer supplemented with a protease inhibitor cocktail (Thermo Fisher Scientific, 1:100 dilution) and ethylenediaminetetraacetic acid (EDTA, 5 mM final concentration), and then incubated at 4°C for 30 min. The samples were sonicated at 4°C, followed by centrifugation at 14,000 × g for 15 min. The supernatant was collected as the extracted protein sample. Protein concentration was determined using a bicinchoninic acid (BCA) assay. For sample preparation, proteins were diluted with 5X sodium dodecyl sulfate (SDS) sample buffer and boiled at 95°C for 5 min. Proteins were separated via sodium dodecyl sulfate-polyacrylamide gel electrophoresis (SDS-PAGE) on 4-12% gradient gels (Thermo Fisher Scientific), followed by transfer onto polyvinylidene fluoride (PVDF) membranes. The membranes were treated with blocking buffer (5% non-fat dry milk in Tris-buffered saline containing 0.1% Tween 20 (TBST)) at RT for 60 min, then incubated with primary antibodies (diluted with 1% non-fat dry milk in TBST) at 4°C overnight. After washing with TBST, the membranes were treated with secondary antibodies (diluted with 1% non-fat dry milk in TBST) at RT for 60 min. The membranes were washed with TBST and treated with Pierce™ ECL Plus Western Blotting Substrate (Thermo Fisher Scientific) at RT for 5 min. Protein bands were visualized using a Gel Doc EZ System (Bio-Rad, Hercules, CA, USA). The antibodies used for WB are listed in Supplemental Table 1.

Data Analysis of Fluorescence Microscopic Images
For comprehensive and unbiased image analysis, a specific region of interest (ROI) measuring 500 µm × 500 µm was designated on each tissue slide. Within this ROI, 100 cells were randomly selected from the entire cell population using the fully automated VIS DIA VisioMorph system (Visiopharm®, Hoersholm, Denmark) to perform un-skewed sampling. The mean fluorescence intensities (MFIs) of RASAL3 and CD8 were calculated for the 100 cells to represent each individual sample. This procedure was repeated across all 112 slides, yielding 112 paired data points. Finally, these per-sample average MFIs were input into GraphPad Prism 10, and Spearman's rank correlation coefficient (ρ) was calculated to evaluate the overall relationship (co-localization) between RASAL3 and CD8 expression levels. To evaluate the macro-scale microenvironmental relationship and clinical relevance of marker expression, digital image analysis (DIA) was performed over a large, standardized ROI measuring 5 mm × 5 mm (25 mm^2) placed on the healthy tissue or primary tumor lesion of each specimen. The Visiopharm® software automatically segmented all individual cells within this expanded region, regardless of cell type, and calculated the global average MFI for both RASAL3 and CD8 across the entire cell population for each sample (n = 112). To assess the functional correlation and potential heterogeneity between the two markers at this tissue scale, a linear regression analysis was performed using the per-sample average MFIs of RASAL3 and CD8, determining the coefficient of determination (R^2) and significance (p-value). Furthermore, to investigate how these primary tumor expression levels of RASAL3 relate to tumor progression, the dataset was stratified based on clinical records into two distinct cohorts: the non-metastasis group and the metastasis group. Differences in the global average MFIs between the two groups, as well as their regression patterns, were statistically analyzed and visualized using GraphPad Prism 10.

Supplementary Tables

Supplementary Table 1. List of antibodies used in this study

Antigen	Conjugation	Clone or product No.	Working Concentration, dilution or dose	Vendor	Use
CD45	BV785	30-F11	0.25 µg/mL	BioLegend	FC
CD3	FITC	17A2	0.25 µg/mL	BioLegend	FC
CD4	APC-Cy7	GK1.5	0.25 µg/mL	BioLegend	FC
CD8	PE-Cy7, PB	53-6.7	0.25 µg/mL	BioLegend	FC
CD11b	FITC, PE-Cy7	M1/70	0.25 µg/mL	BioLegend	FC
Ly-6G	PB	1A8	0.25 µg/mL	BioLegend	FC
Ly-6C	APC	HK1.4	0.25 µg/mL	BioLegend	FC
B220	PE-Cy7	RA3-6B2	0.25 µg/mL	BioLegend	FC
F4/80	BV650	BM8	0.25 µg/mL	BioLegend	FC
CD80	FITC	16-10A1	0.25 µg/mL	BioLegend	FC
CD86	APC	GL-1	0.25 µg/mL	BioLegend	FC
I-A^b	PB	AF6-120.1	0.25 µg/mL	BioLegend	FC
IFN-γ	PE	XMG1.2	0.5 µg/mL	BioLegend	FC
TNF	APC	MP6-XT22	0.5 µg/mL	BioLegend	FC
Granzyme B	PerCP-Cy5.5	QA16A02	0.5 µg/mL	BioLegend	FC
IL-6	PE	MP5-20F3	0.5 µg/mL	BioLegend	FC
IL-12/IL-23p40	PerCP-Cy5.5	C15.6	0.5 µg/mL	BioLegend	FC
TRP-2-H-2K^b-Tetramer	APC	-	0.5 µg/mL	MBL	FC
SIINFEKL-H-2K^b-Tetramer	APC	-	0.5 µg/mL	MBL	FC
SINNFEKL-H-2K^b	APC		0.5 µg/mL	BioLegend	FC
PD-1	PE	29F.1A12	0.25 µg/mL	BioLegend	FC
CD16/CD32	-	2.4G2	0.5 µg/mL	BioLegend	FC
RASAL3	-	-	1:100	Homemade	FC
TOX	PE	W17072A	0.5 µg/mL	BioLegend	FC
PD-1	-	10F.9G2™	200 µg/dose	Bio X Cell	ICB
Rat IgG2b,k (Isotype)	-	-	200 µg/dose	Bio X Cell	ICB
RASAL3	-	PA5-60358	1:100	Thermo	IF

CD8	-	BX50036-C3	1:100	Biolynx	IF
Goat Anti-Rabbit IgG H&L	FITC	ab6717	1:1000	Abcam	IF
Goat Anti-Rabbit IgG H&L	PE	Ab72465	1:1000	Abcam	IF
RASAL3	-	orb3095101	1:1000	Biorbyt	WB
Actin	-	BA3R	1:1000	Thermo	WB
Goat anti-Rabbit IgG (H+L)	HRP	65-6120	1:10000	Thermo	WB
Goat anti-mouse Ig (H+L)	HRP	31430	1:10000	Thermo	WB

Supplementary Table 2. Sample and patient information

No.	ID	Sex	Age	Survival (Days after diagnosis)	Sample origine	Metastasis	Location (Original)	Location (Metastasis)
1	L01A0148	F	62		Skin	-	Forehead	
2	L01A0227	M	66		Skin	-	Medial side of the left big toe	
3	L01A0138	M	73		Skin	-	Palmar side of the right hand	
4	L01A0161	F	81		Skin	-	Mole on the right arm	
5	L01A0162	F	87		Skin	-	Mons pubis and clitoris region	
6	L01A0163	F	87		Skin	-	Abdominal wall nodule	
7	L01A0164	M	55		Skin	-	Distal phalanx of the right big toe	
8	L01A0165	F	65		Skin	-	Skin of the right heel	
9	L01A0166	M	60		Skin	-	Skin of the left elbow	
10	L01A0167	F	52		Skin	-	Groin	
11	L01A0229	M	28		Skin	-	Buttock	
12	L01A0230	M	83		Skin	-	Right forefoot and right big toe	
13	L01A0232	F	78		Skin	-	3rd and 4th toes of the right foot	
14	L01A0233	F	36		Skin	-	Left lower leg, left sole	
15	L01A0234	M	57		Skin	-	Right sole	
16	L01A0312	M	65		Skin	-	Right thumb	
17	L01A0313	M	58		Skin	-	Right armpit	
18	L01A0314	M	40		Skin	-	Left buttock	
19	L01A0315	F	73		Skin	-	Right sole	
20	L01A0316	F	73		Skin	-	Left heel	
21	L01A0317	M	65		Skin	-	Left sole	
22	L01A0318	M	54		Skin	-	Left heel	
23	L01A0319	F	67		Skin	-	Right sole	
24	L01A0320	F	93		Skin	-	Right armpit	
25	L01A0321	F	70		Skin	-	Right thigh	
26	L01A0322	F	77		Skin	-	Left sole	
27	L01A0325	M	73		Skin	-	Left finger	
28	L01A0330	M	33		Skin	-	Subcutaneous tissue of the left thigh	
29	L01A0331	F	71		Skin	-	Right foot	
30	L01A0332	F	43		Skin	-	Right arm	
31	L01A0333	M	64		Skin	-	Left upper arm, left elbow, and left little finger	
32	L01A0334	F	74		Skin	-	Right sole	
33	L01A0335	F	45		Skin	-	Right lower leg	
34	L01A0336	M	63		Skin	-	Groin and back	
35	L01A0337	M	59		Skin	-	Chest wall	
36	L01A0338	M	49		Skin	-	Left thumb	
37	L01A0339	F	78		Skin	-	Left index finger	
38	L01A0340	M	69		Skin	-	Right sole	
39	L01A0341	F	45		Skin	-	Right sole	
40	L01A0344	F	45		Skin	-	Right thigh	
41	L01A0352	F	69		Skin	-	Right foot	
42	L01A0357	M	61		Skin	-	Dorsum of the right foot	
43	L01A0372	F	62		Skin	-	Right index finger	
44	L01A0374	F	81		Skin	-	Right lower extremity	
45	L01A0383	M	43		Skin	-	Left foot	
46	L01A0384	M	33		Skin	-	Right thumb	
47	L01A0385	F	50		Skin	-	Left thumb nail bed	
48	L01A0388	M	30		Skin	-	Neck	
49	L01A0391	M	74		Skin	-	Left auricle	
50	L01A0389	F	72		Skin	-	Anal orifice	
51	D08A0578	M	77		Skin	-	Lower esophagus	
52	D08A1797	F	39		Skin	-	Lower esophagus	
53	D08A3438	M	47		Skin	-	Esophagus	
54	D09B1503	M	66		Skin	-	Gastric body and fundus	
55	D99A0171	M	72		Skin	-	Small intestine	
56	D99A0198	M	59		Skin	-	Small intestine	
57	D15A6203	F	30		Skin	-	Left colon	
58	D15A7336	F	55		Skin	-	Right colon and part of the small intestine	
59	D16A6362	F	68		Skin	-	Rectum	
60	D16A6599	M	79		Skin	-	Rectum	

No.	ID	Sex	Age	Survival (Days after diagnosis)	Sample origine	Metastasis	Location (Original)	Location (Metastasis)
61	D16A6601	M	54		Skin	-	Rectum	
62	D17A0023	F	53		Skin	-	Anal canal	
63	D17A0024	F	63		Skin	-	Anorectum	
64	D17A0025	F	82		Skin	-	Anal canal, Rectum	
65	D01A0009	F	77		Skin	-	Left lower lip	
66	D03A0007	M	60		Skin	-	Right palatal mucosa	
67	D03A0008	F	56		Skin	-	Palate, Oral mucosa	
68	D04A0011	M	44		Skin	-	Right upper gum	
69	E01A0107	F	79		Skin	-	Nasal cavity tissue, left ethmoid sinus tissue, right nostril	
70	E01A0109	M	54		Skin	-	Bilateral nasal cavities	
71	E01A0115	F	50		Skin	-	Left nasal cavity	
72	E01A0120	F	72		Skin	-	Left nasal cavity	
73	E01A0121	F	65		Skin	-	Right maxillary sinus	
74	E01A0122	F	68		Skin	-	Nasopharynx	
75	E01A0123	M	78		Skin	-	Left nasal cavity	
76	E01A0124	F	65		Skin	-	Right maxillary sinus	
77	E01A0125	M	79		Skin	-	Left nasal cavity	
78	E01A0132	F	77		Skin	-	Right nasal cavity	
79	E01A0133	F	72		Skin	-	Left nasal cavity and paranasal sinuses	
80	E01A0134	M	74		Skin	-	Right nasal cavity	
81	M03A0004	M	63		Skin	-	Within the right orbit, right lower eyelid	
82	M03A0005	M	76		Skin	-	Right eyeball	
83	M03A0006	M	92		Skin	-	Right eyelid	
84	M03A0008	F	75		Skin	-	Left conjunctiva	
85	M02A0003	F	70		Skin	-	Left eyeball	
86	M02A0004	M	75		Skin	-	Left eyeball	
87	K02A0044	M	45		Skin	-	Abdominal wall	
88	K02A0104	M	76		Skin	-	Left abdominal wall	
89	L02A0068	M	40		Skin	-	Right arm	
90	L02A0121	M	52		Skin	-	Left buttock	
91	J04A0016	F	63		Skin	-	Middle-to-upper segment of the vaginal wall	
92	J04A0017	F	60		Skin	-	Vagina	
93	P01A0412	F	82		Skin	-	Left temporal region	
94	J07A3053	F	67		Skin	-	Right breast	
95	L01A0121	M	54		Skin	-	Left mandible	
96	H02A0123	M	80	1022	Skin	+	Right armpit	Right inguinal lymph node
97	H02A0124	M	66	881	Skin	+	Left arm	Left inguinal lymph node
98	H02A0125	M	81	321	Skin	+	Neck	Right inguinal lymph node and soft tissue
99	H02A0127	M	61	182	Skin	+	Left abdominal wall	Left inguinal lymph node
100	H02A0394	F	46	102	Skin	+	Rectum	Pelvic lymph nodes
101	H02A0479	M	48	332	Skin	+	Right thumb	Right axillary lymph node
102	H02A0480	M	63	2681	Skin	+	Left sole	Left thigh lymph node
103	H02A0481	M	59	872	Skin	+	Rectum	Right inguinal lymph node
104	H02A0482	M	71	452	Skin	+	Right sole	Right inguinal lymph node
105	H02A0484	M	49	2861	Skin	+	Left nasal cavity	Left shoulder lymph node
106	H02A0485	F	84	1053	Skin	+	Right nasal cavity	Left axillary lymph node
107	H02A0494	M	71	281	Skin	+	Oral mucosa	Left parotid lymph node
108	H02A0523	M	51	3812	Skin	+	Right lower extremity	Left inguinal lymph node
109	P01A0411	F	52	1856	Skin	+	Left arm	Right Parietal Lobe of the brain.
110	P01A0463	M	50	2863	Skin	+	Right eyeball	Right Basal Ganglia region of the brain
111	L01A0160	M	40	216	Skin	+	Right sole	Buttocks
112	E05A4843	M	66	782	Skin	+	Right arm	Right lung

Supplementary Table 3. Primer sequences for genotyping PCR

Target	Primers
RASAL3-flox	Forward: 5’-TGCTACTAGGCCTACCTTTG-3’<br>Reverse1: 5’-TAGTGTCCAAGAAAGCCAGC-3’
CD8-Cre	Transgene forward: 5’- CAATGGAAGGAAGTCGTGGT-3’<br>WT forward: 5’-CACACATGCAAGTCTAAATCAGG-3’<br>Common reverse: 5’-TGGGATTTACAGGGCATACTG-3’

Supplemental Figures

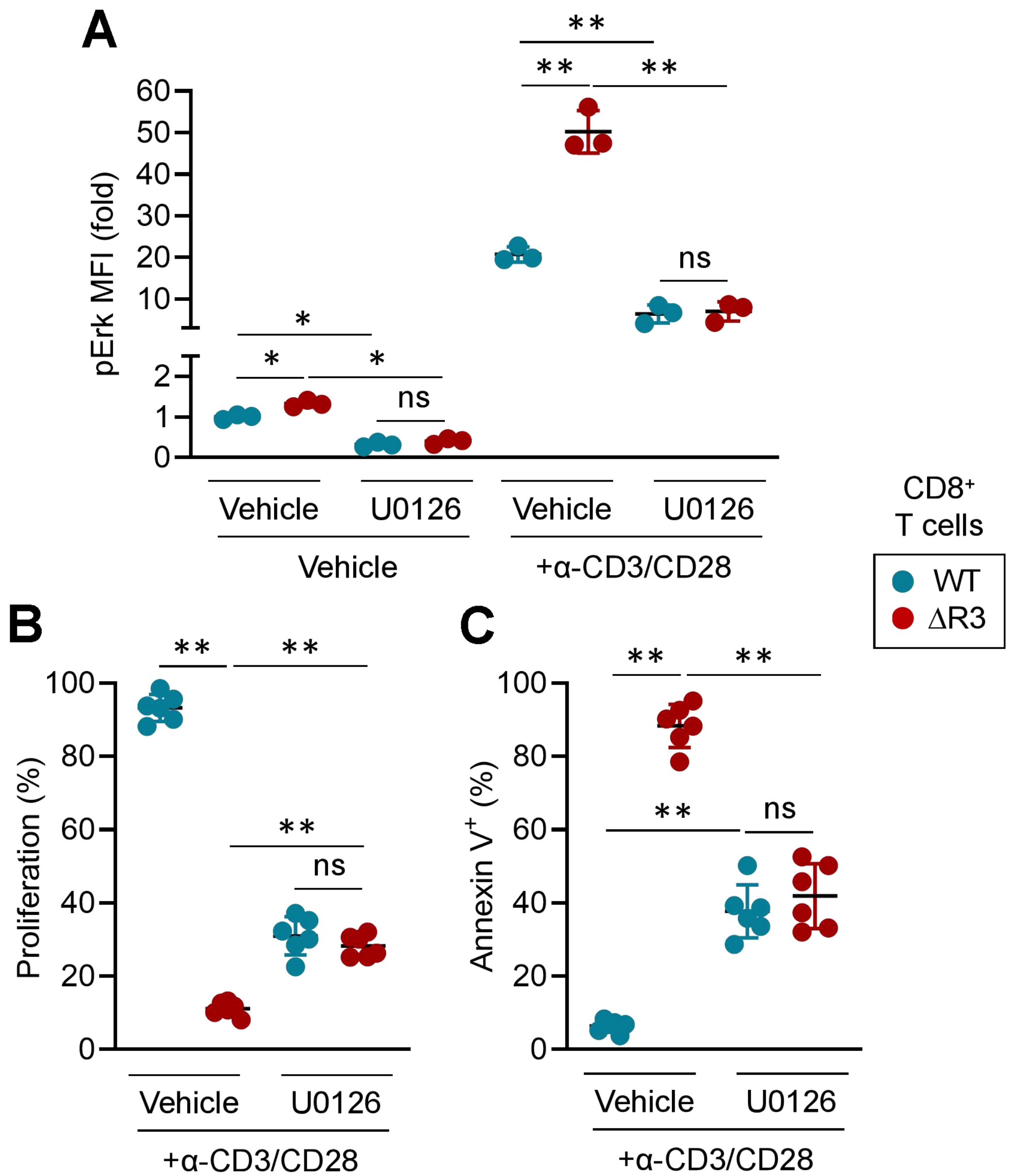

Supplemental Figure 1. MEK inhibition attenuates Ras signaling hyperactivation, rescuing cell proliferation and inhibiting apoptosis in RASAL3-deficient CD8⁺ T cells

Splenic CD8⁺ T cells were isolated from WT or RASAL3-KO mice (WT or ΔR3 CD8⁺ T cells). For the proliferation assay, cells were stained with CFSE prior to experiments. CD8⁺ T cells were pre-incubated with vehicle or U0126 (MEK inhibitor, 10 μM) at 37°C for 60 min, followed by anti-CD3/CD28 microbead stimulation. After incubation, pERK levels, cell proliferation, and Annexin V levels were analyzed by flow cytometry. A) Cumulative pERK levels in CD8⁺ T cells. B) Cumulative proliferation rates of CD8+ T cells after 72 h of incubation. C) Cumulative Annexin V levels of CD8+ T cells after 72 h of incubation. Cumulative data are shown as the mean ± standard deviation (SD) of six (Figure 1B and G) to eight (others) samples from three independent experiments. All MFI fold-change values were calculated by setting the

mean MFI value of the control group to 1. Welch's t-test or one-way ANOVA was used to determine statistical significance. *$p < 0.05$ and **$p < 0.01$; ns, not significant.

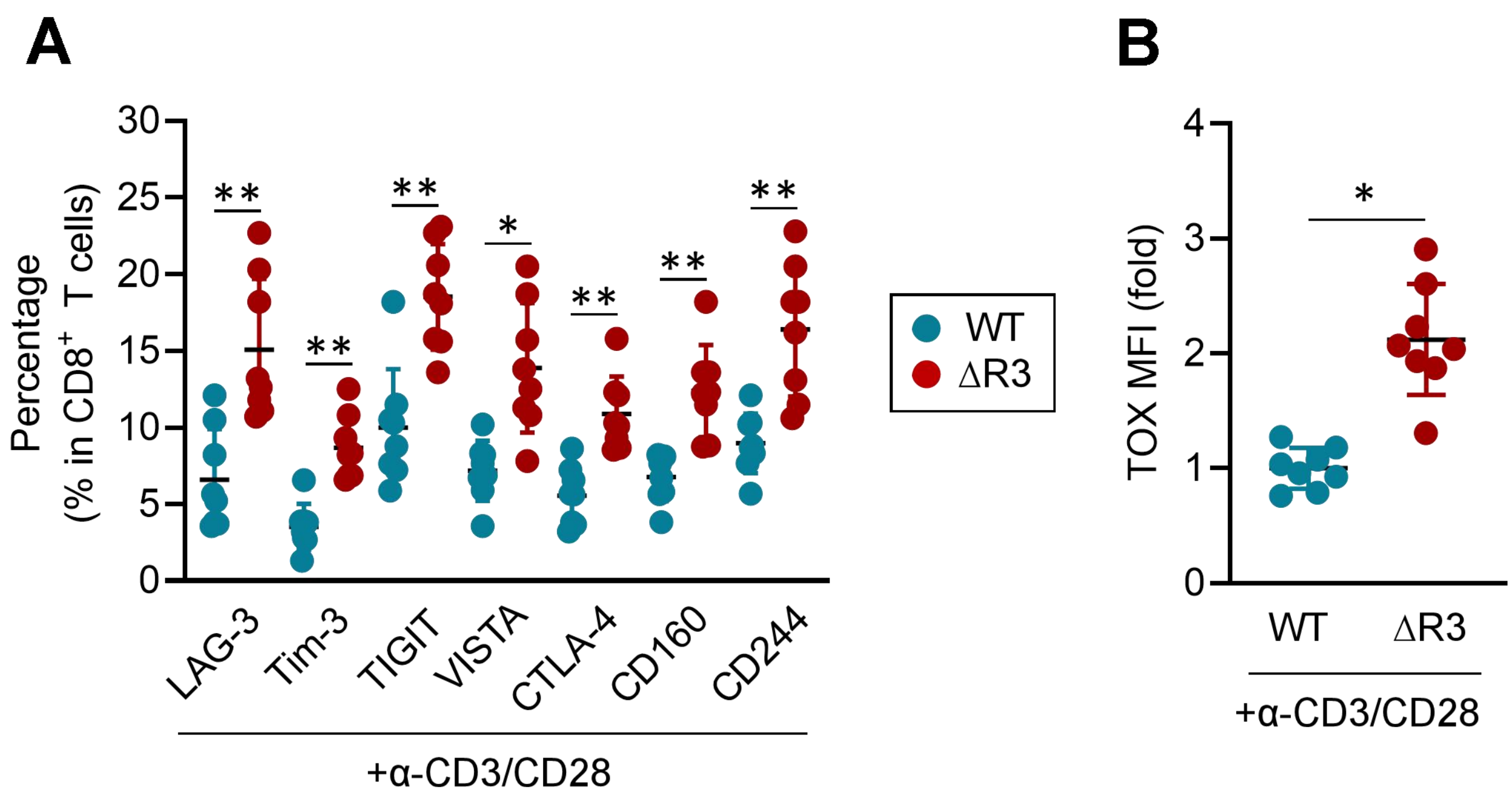

Supplemental Figure 2. RASAL3 deficiency promotes an exhausted phenotype in CD8+ T cells upon TCR stimulation

Splenic CD8$^+$ T cells were isolated from WT or RASAL3-KO mice (referred to as WT or ΔR3 CD8$^+$ T cells) and stimulated with anti-CD3/CD28 microbeads at 37°C for 72 h. The expression of inhibitory checkpoint molecules (ICMs) and TOX was analyzed by flow cytometry. A) Cumulative percentage values of ICM-expressing CD8$^+$ T cells. B) Cumulative MFI values (fold change) of TOX in CD8$^+$ T cells. Cumulative data are shown as the mean ± standard deviation (SD) of eight samples from three independent experiments. All MFI fold-change values were calculated by setting the mean MFI value of the control group to 1. Welch's t-test or one-way ANOVA was used to determine statistical significance. *$p < 0.01$ and **$p < 0.001$; ns, not significant.

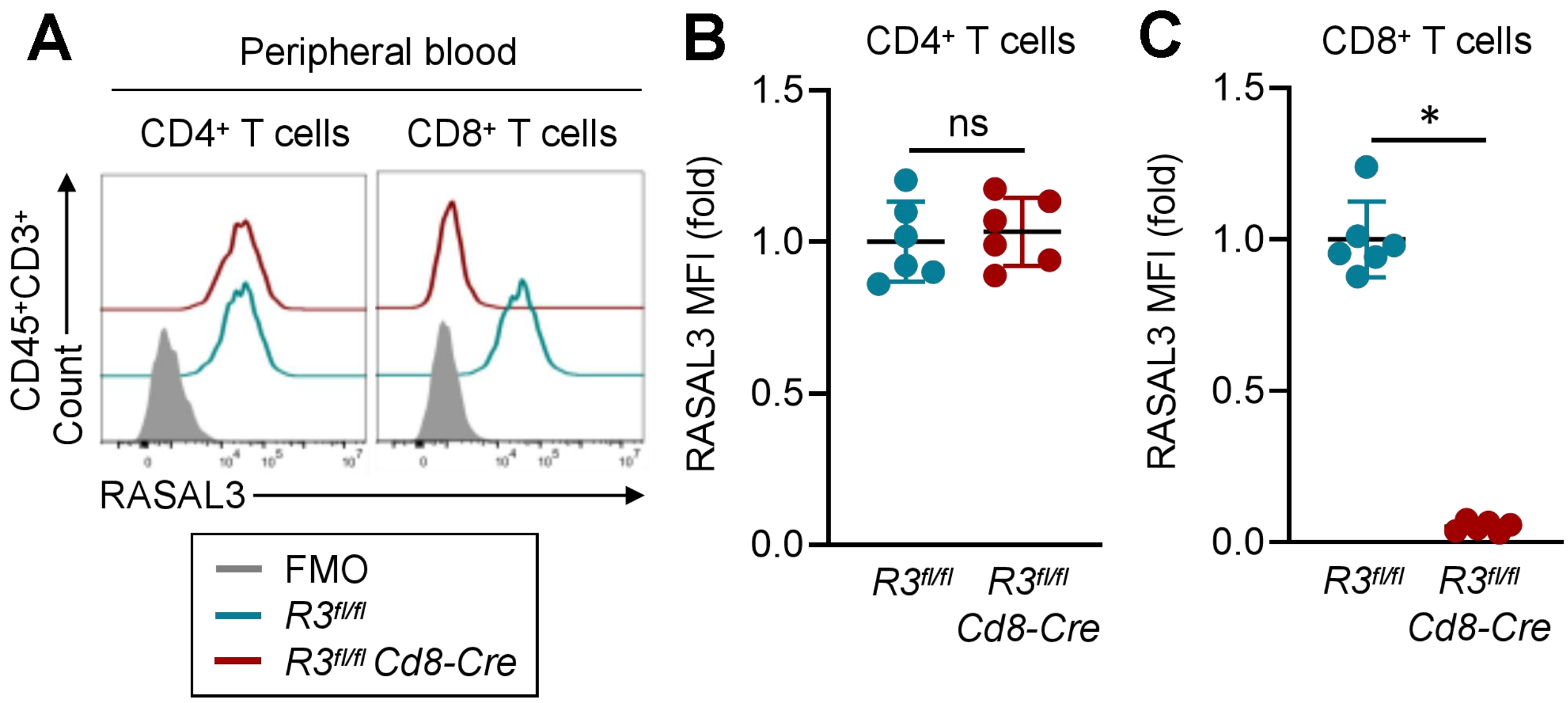

Supplementary Figure 3. CD8⁺ T cell specific RASAL3 depletion in *R3^fl/fl^Cd8-Cre* mice

RASAL3 protein expression was screened in peripheral blood circulating $CD4^+$ T cells and $CD8^+$ T cells by flow cytometry. Representative histograms (A), cumulative RASAL3 MFI (fold change) of $CD4^+$ T cells (B) and $CD8^+$ T cells (C) are presented, respectively. All MFI values are represented as fold changes (the average value of WT was used for baseline=1). A Mann-Whitney U test was used to analyze data for significant differences, and *$p < 0.001$ was considered as significant difference. ns; not significant.

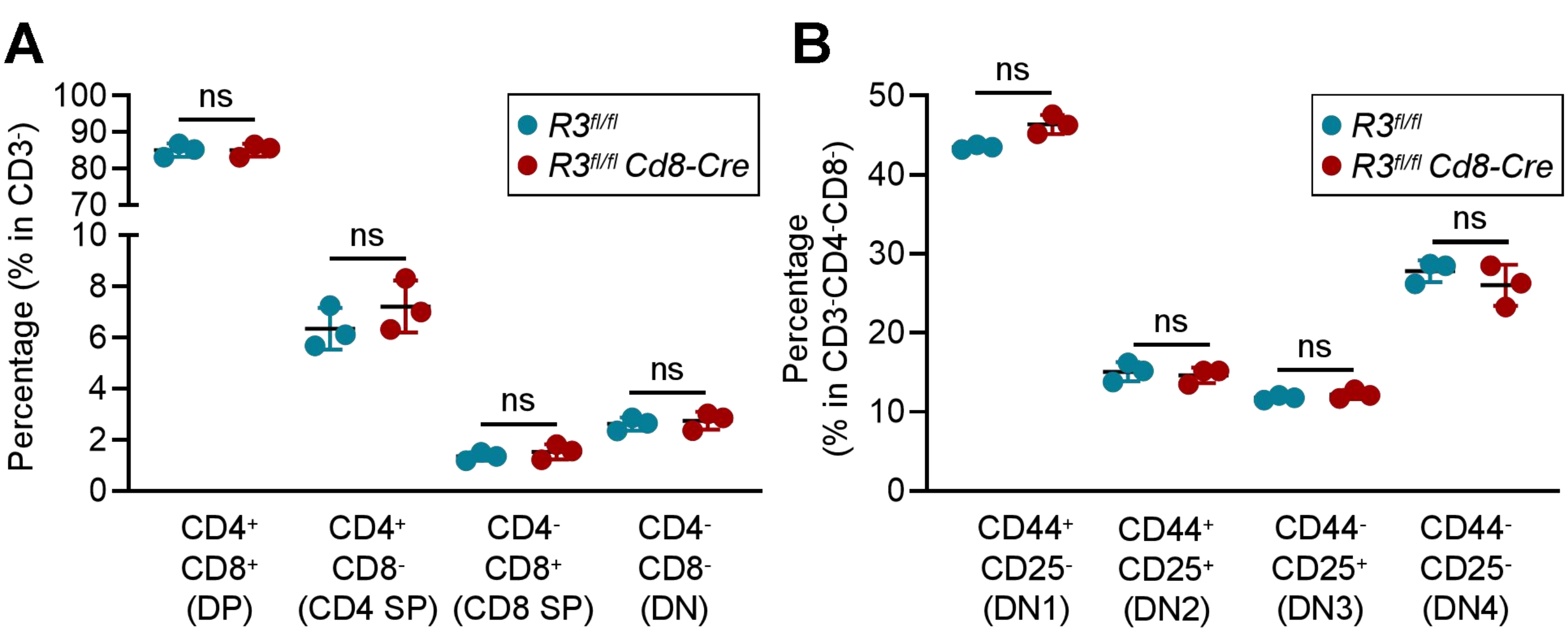

Supplementary Figure 4. Normal thymic T cell development of *R3^fl/fl^Cd8-Cre* mice

WT or *R3^fl/fl^-Cd8-Cre* mice were subjected to analyze T cell development in thymus. A) The frequencies of double positive (DP; $CD4^+CD8^+$), CD4 single positive (SP; $CD4^+CD8^-$), CD8 SP ($CD4^-CD8^+$) and double negative (DN; $CD4^-CD8^-$) cells in $CD45^+CD3^-$ population. frequencies in peripheral lymphoid organs by flow cytometry. B) The frequencies of DN1 ($CD44^+CD25^-$), DN2 ($CD44^+CD25^+$), DN3 ($CD44^-CD25^+$) and DN4 ($CD44^-CD25^-$) cells in the DN population. The cumulative data are shown as mean ± SD values of three samples. A Mann-Whitney U test was used to analyze data for significant differences. ns; not significant.

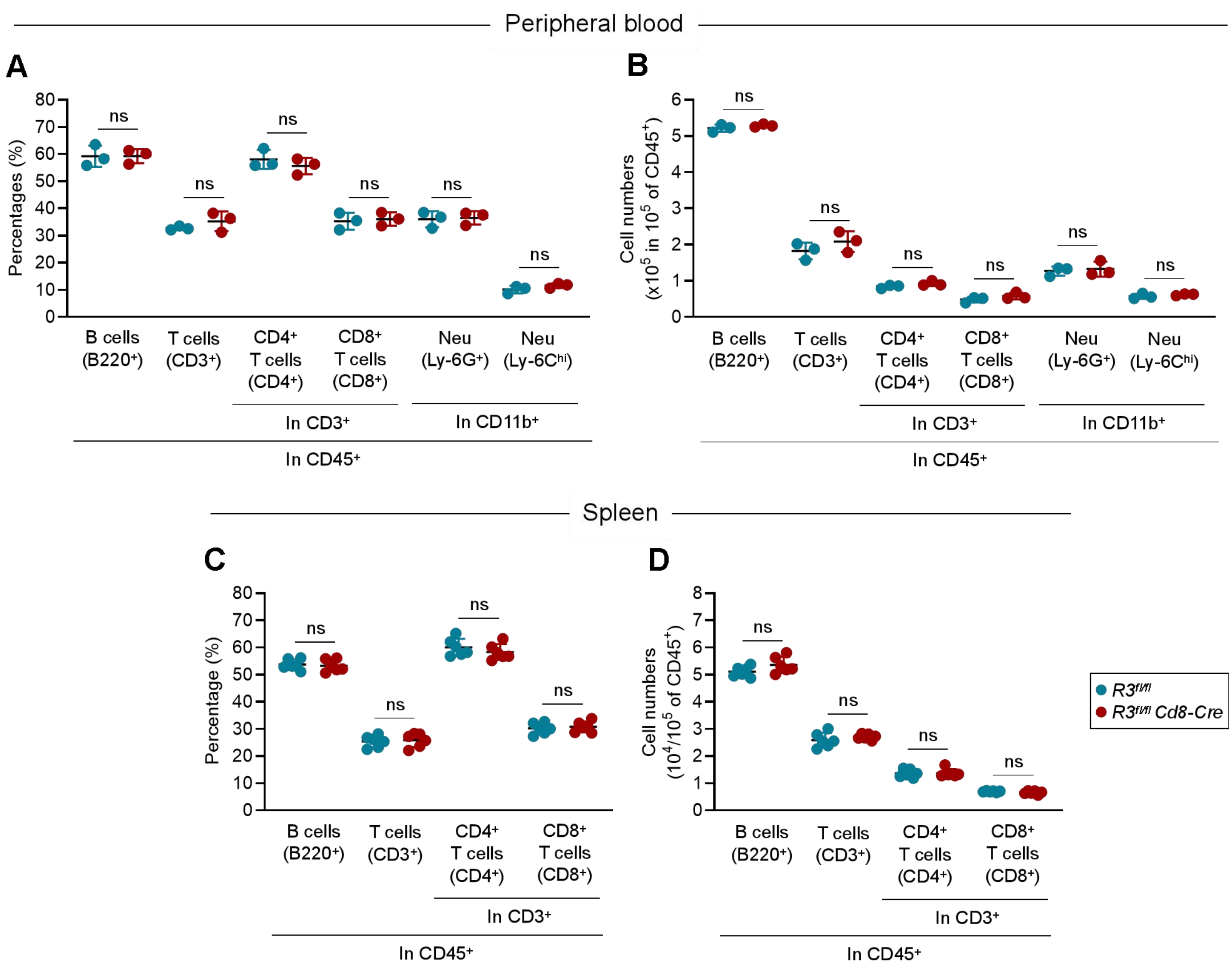

Supplementary Figure 5. Normal lymphoid and myeloid populations in *R3^fl/fl^Cd8-Cre* mice

*R3^fl/fl^* or *R3^fl/fl^Cd8-Cre* mice were subjected to analyze the frequencies and cell numbers of lymphoid and myeloid populations in peripheral blood and spleen using flow cytometry. Each population was identified according to surface marker expression as follows: B cells ($B220^+$ in $CD45^+$), T cells ($CD3^+$ in $CD45^+$), $CD4^+$ T cells ($CD3^+CD4^+$ in $CD45^+CD3^+$), $CD8^+$ T cells ($CD3^+CD8^+$ in $CD45^+$), neutrophils ($CD11b^+Ly\text{-}6G^{+/hi}Ly\text{-}6C^+$ in $CD45^+$) and monocytes ($CD11b^+Ly\text{-}6G^-Ly\text{-}6C^{hi}$ in $CD45^+$). Cumulative data are shown as mean ± SD values of three (A, B) or six samples (C, D). Welch's t-test was used to analyze data for significant differences. ns; not significant.

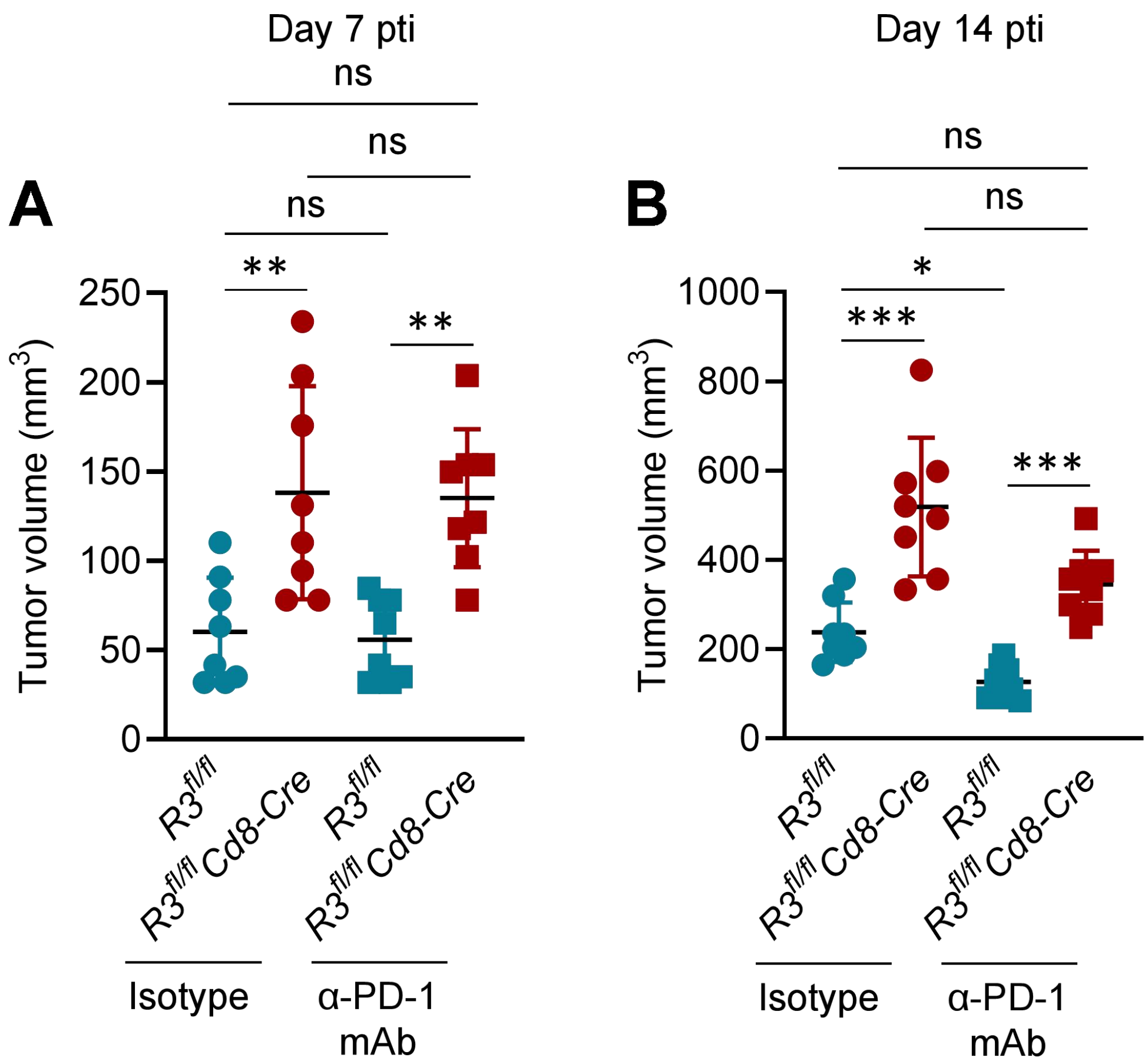

Supplementary Figure 6. Tumor volumes in B16-F10-inoculated mice

WT or *R3*$^{fl/fl}$*-Cd8-Cre* mice were inoculated with B16-F10 via s.c. injection, and then tumor volumes were measured on day 7 and 14 pti. The cumulative data are shown as mean ± SD values for six samples from two independent experiments. Two-way ANOVA was used to analyze data for significant differences. *p<0.05, **p < 0.01 and ***p<0.001, ns; not significant.

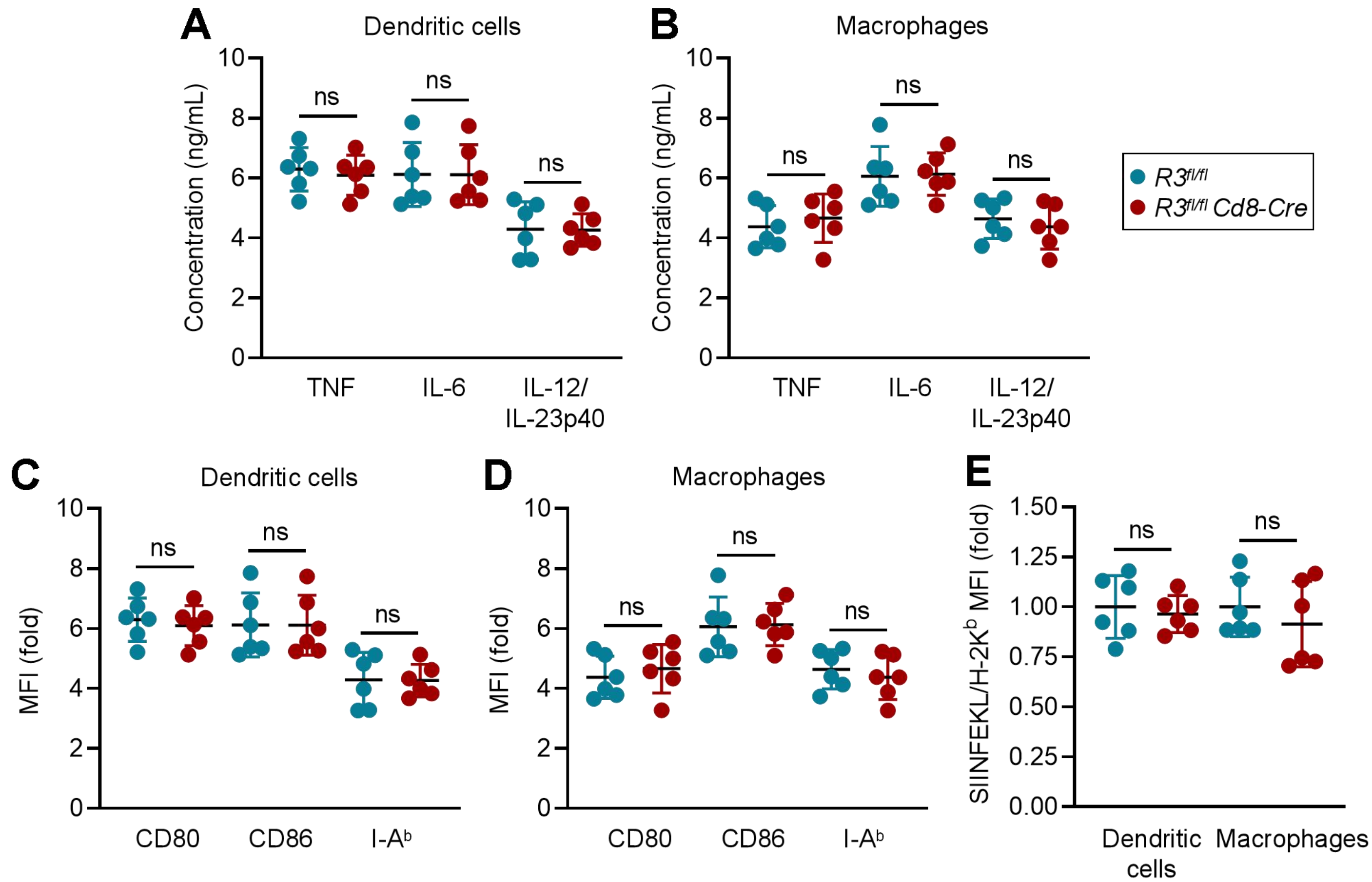

Supplementary Figure 7. Normal APC function in *R3fl/flCd8-Cre* mice

Dendritic cells (BMDCs) or macrophages (TPMs) derived from WT or *R3fl/fl-Cd8-Cre* mice were subjected to investigate APC function by *in vitro* assays. A-B) Cytokine production assay. The BMDCs (A) or TPMs (B) were stimulated with LPS at 37°C for 16 h. The cytokine (TNF, IL-6 and IL-12/IL-23p40) concentrations in the cultured medium were measured by ELISA. C-D) Cellular activation assay. The BMDCs (C) or TPMs (D) were stimulated with LPS at 37°C for 16 h. The cell surface expressions of CD80, CD86 and MHC class II (I-A^b) were analyzed by flow cytometry. E) Antigen processing and presentation assay. The BMDCs or TPMs were cultured with OVA at 37°C for 6 h. The cell surface expression of the OVA-originated peptide antigen-MHC class I complex (SIINFEKL/H-$2K^b$) was analyzed by flow cytometry. The cumulative data are shown as mean ± SD values of six samples from two independent experiments. All MFI values are represented as fold changes (the average value of WT was used for baseline=1). A Mann-Whitney U test was used to analyze data for significant differences. ns; not significant.

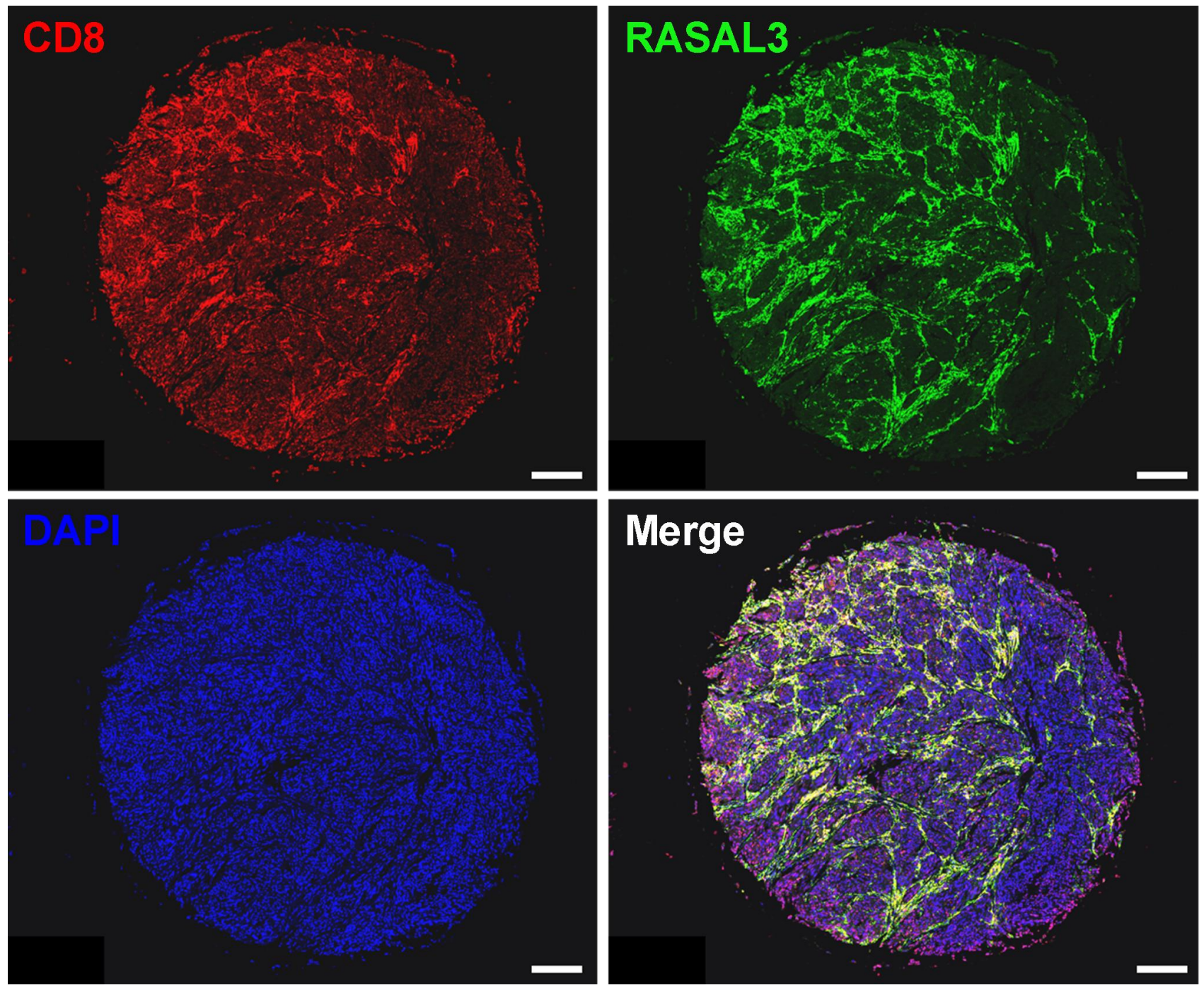

Supplementary Figure 8. Immunofluorescence analysis of human melanoma tissue
Human melanoma tissue sections were stained with anti-CD8 and anti-RASAL3 antibodies, followed by incubation with fluorochrome-conjugated secondary antibodies. Quantitative analysis was performed using digital image analysis (DIA) with the fully automated VisioMorph system (Visiopharm®, Hørsholm, Denmark). Scale bar = 200 µm.

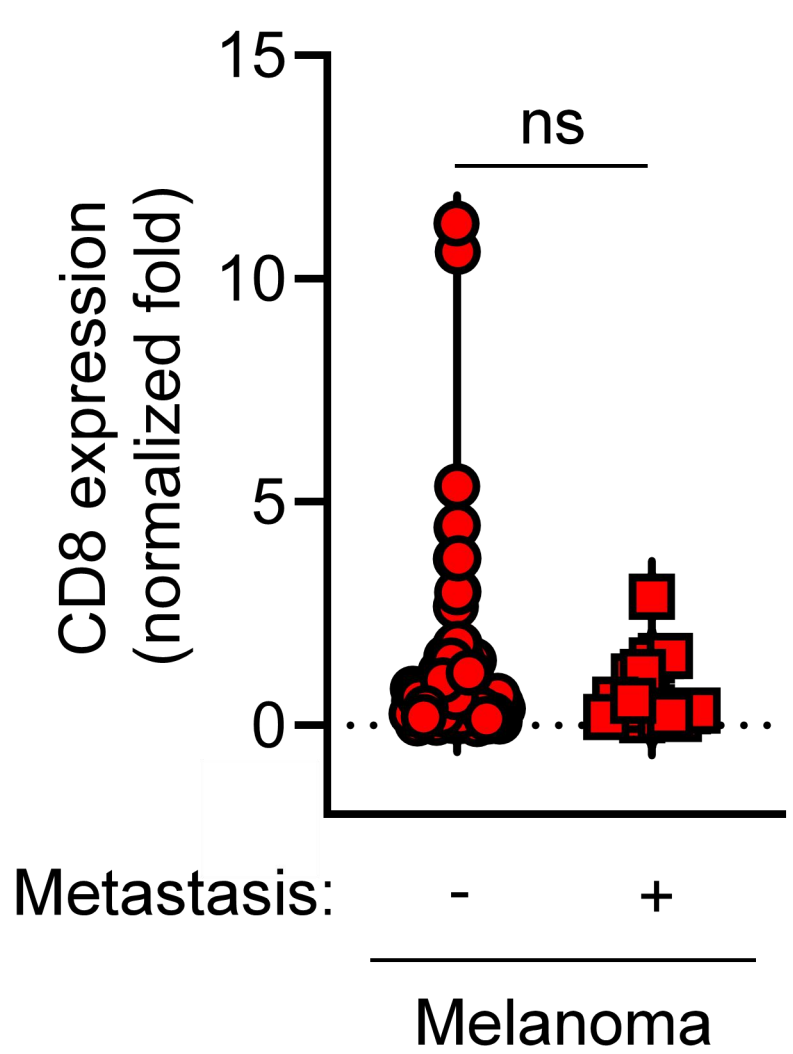

Supplemental Figure 9. CD8 intensity in melanoma tissues
CD8 intensities in primary skin melanoma were compared between patients without metastasis (-) (n=95) and patients with metastasis (+) (n=17). Cumulative data are shown as the mean ± SD of all analyzed samples. Mann-Whitney U test was used to determine statistical significance. *$p < 0.05$.
